# Global, regional, and national epidemiology of Intracerebral Hemorrhage among women of childbearing age (1990–2021): incidence, mortality, DALYs, and risk factor analysis

**DOI:** 10.3389/fneur.2025.1602507

**Published:** 2025-08-05

**Authors:** Yuan Li, Wei Yang, Jixiang Xu, Guoming Pu, Sujun Liu, Chunmei Chen, Xiufang Che, Hua Huang, Hongjiang Pu

**Affiliations:** ^1^Department of Neurology, Dazhou Central Hospital, Dazhou, Sichuan, China; ^2^General Department of Hyperbaric Oxygen, The Second People's Hospital of Hefei, Hefei Afliated Hospital of Anhui Medical University, Hefei, Anhui, China; ^3^Department of Obstetrics and Gynecology, People's Hospital of Tongchuan District, Dazhou, Sichuan, China; ^4^The Third Affiliated Hospital of Kunming Medical University, Peking University Cancer Hospital Yunnan Hospital, Yunnan Cancer Hospital, Kunming, China

**Keywords:** Intracerebral Hemorrhage, WCBA, global burden of disease, disease burden, DALYs, GBD 2021

## Abstract

**Background and Objectives:**

Intracerebral Hemorrhage (ICH) is a serious cerebrovascular condition that poses a significant health risk to women of childbearing age (WCBA) globally. However, research specifically focusing on the global impact and trends of ICH among WCBA is limited. This study aims to present data on the burden of ICH among WCBA from 1990 to 2021 across 204 countries and regions, with an emphasis on health disparities, to inform more effective prevention and intervention strategies.

**Methods:**

Using data from the Global Burden of Disease Study (GBD 2021), this study evaluates the global, regional, and national impact of ICH from 1990 to 2021, focusing on incidence, prevalence, mortality, and disability-adjusted life years (DALYs). Temporal trends were analyzed using the estimated annual percentage change (EAPC), and health disparities were assessed using the slope index of inequality. Frontier analysis was employed to identify achievable outcomes based on development levels, and the Bayesian age-period-cohort model was used to project future disease burden trends.

**Results:**

In 2021, the global burden of ICH among WCBA decreased compared to 1990. There were 231,888 cases (95% UI: 163,211–320,867) and 94,223 deaths (95% UI: 82,659–106,578), with age-standardized incidence and death rates of 11.493 (95% UI: 8.073–15.925) and 4.628 (95% UI: 4.059–5.237) per 100,000 people, respectively. DALYs lost totaled 5,040,596 years (95% UI: 4,432,534–5,654,102), with a DALY rate of 249.334 (95% UI: 222.411–277.622) per 100,000 people. The burden was linked to the Socio-Demographic Index (SDI), with lower SDI regions experiencing increasing burden, while higher SDI regions saw a decline. Regional disparities were observed, and certain factors like environmental particulate pollution showed an upward trend. Projections for 2035 indicate continued decline in both ASDR and ASIR.

**Conclusion:**

ICH in women of reproductive age remains a significant global public health issue. While the overall burden has declined, disparities persist, especially in regions with lower development. Addressing these disparities requires targeted public health interventions, optimized healthcare resource distribution, improved healthcare infrastructure, and health education, in line with the WHO's 2030 health objectives.

## 1 Introduction

Intracerebral Hemorrhage (ICH) is the second most prevalent form of stroke, following ischemic stroke, and is associated with high rates of mortality and disability, particularly in women of childbearing age (WCBA), which has notable clinical relevance (1, 2). While ICH is more common among older individuals, WCBA face a higher risk due to their unique physiological traits and hormonal changes, especially during pregnancy and the postpartum period (3). The impact of ICH on WCBA extends beyond the individual, affecting the health of the fetus and family, and contributing to substantial social and economic burdens (4, 5). As a result, examining ICH in this demographic is crucial for public health.

The Global Burden of Disease (GBD) database collects data on disease incidence, mortality, and disability-adjusted life years (DALYs) for various health conditions (6). By tracking disease burden trends and disparities across populations, it offers valuable insights for shaping health policies, distributing resources, and designing interventions (7). However, specific data on ICH among WCBA is still scarce. In 2019, over 1.5 million people worldwide were affected by ICH, resulting in more than 400,000 deaths and millions of DALYs lost (8). Although recent advancements in medicine have decreased both the incidence and mortality of ICH, the global burden remains significant, with notable regional variations (9).

Due to physiological and hormonal fluctuations, WCBA are at heightened risk of severe ICH, particularly during pregnancy, childbirth, and the postpartum period (10). Hypertensive disorders of pregnancy are the primary risk factor for pregnancy-associated Intracerebral Hemorrhage (ICH), with preeclampsia increasing risk through impaired cerebral autoregulation and endothelial dysfunction. Eclampsia causes ICH in 0.2%−2.8% of cases through severe blood pressure surges (11). HELLP syndrome increases risk via thrombocytopenia and microvascular dysfunction. Additional factors include gestational hypertension, peripartum cardiomyopathy, and choriocarcinoma (12). Approximately 50% of pregnancy-associated ICH occurs within 6 weeks postpartum due to hemodynamic stress and persistent vascular alterations. Pregnancy's 40%−50% increase in plasma volume and cardiac output, combined with hormonal changes affecting vascular tone and coagulation, creates multifactorial mechanisms underlying ICH risk despite protective prothrombotic states (13).

Despite some progress in medical research, epidemiological data on ICH in WCBA remains limited, with few comprehensive global studies available. Therefore, investigating ICH in this population is essential for improving the understanding of its trends and clinical manifestations, revealing regional disparities, and providing critical data for developing more effective prevention strategies, treatments, and public health policies.

## 2 Materials and methods

### 2.1 Data sources and disease definition

This study utilized data from the Global Burden of Disease (GBD) 2021 study, which provides the most recent epidemiological estimates for 371 diseases and injuries across 21 GBD regions and 204 countries and territories from 1990 to 2021 (14). The dataset is publicly accessible through the Global Health Data Exchange (https://ghdx.healthdata.org/gbd-2021/sources) (15). The research focuses on the epidemiological impact of ICH among WCBA by analyzing prevalence, incidence, mortality, and disability-adjusted life years (DALYs). Data were obtained from the Institute for Health Metrics and Evaluation (IHME), ensuring consistency and cross-national comparability. The definition of ICH adheres to the International Classification of Diseases, 10th Revision (ICD-10). Further details on GBD data sources, methodologies, statistical models, and study design are well documented in previous reports (16, 17). All disease and injury data related to ICH in the GBD database were systematically integrated to maintain accuracy and reliability.

### 2.2 Study population

This study analyzed WCBA diagnosed with ICH between 1990 and 2021. WCBA is defined as females aged 15–49 years, following World Health Organization (WHO) standards (18). The data from GBD 2021 were examined to determine the incidence and burden of ICH among WCBA across different regions.

### 2.3 Sociodemographic Index and disability-adjusted life years

To assess the level of development in each country and region, this study employed the Sociodemographic Index (SDI) developed by the Institute for Health Metrics and Evaluation (IHME). SDI is a composite index ranging from 0 to 1, calculated as the geometric mean of total fertility rate under age 25, mean years of education for individuals aged 15 and older, and per capita income. In GBD 2021, SDI values were rescaled from 0 to 100, with higher values indicating greater socioeconomic development. Based on SDI scores, 204 countries and territories were classified into five categories: low, low-middle, middle, high-middle, and high SDI regions (19). The burden of ICH among WCBA was quantified using disability-adjusted life years (DALYs), which measure the total number of healthy life years lost due to disease. DALYs comprise years of life lost (YLLs) due to premature mortality and years lived with disability (YLDs), calculated as DALYs = YLLs + YLDs. Following GBD methodology, all calculations were performed 500 times to generate sampled estimates, with final values representing the mean of these 500 samples and 95% confidence intervals (CIs) derived from the 2.5th and 97.5th percentiles of the sampled values. Uncertainty was accounted for at each step of the estimation process (13). Since GBD estimates do not directly attribute deaths to ICH, YLLs were calculated based on ICH-related mortality rates and expected life expectancy.

### 2.4 Age-standardized rates and estimated annual percentage changes

To ensure comparability across regions and time periods, we employed age-standardized rates (ASRs) using direct standardization with the WHO Global Standard Population (2000–2025). Age-specific rates were extracted for seven WCBA age groups (15–19, 20–24, 25–29, 30–34, 35–39, 40–44, and 45–49 years), with corresponding WHO standard weights renormalized within the 15–49 age range. The final age-specific weights were: 15–19 years (0.127), 20–24 years (0.146), 25–29 years (0.153), 30–34 years (0.156), 35–39 years (0.156), 40–44 years (0.138), and 45–49 years (0.124).

ASRs were calculated using: ASR = (∑(*r*_*i*_ × ω_*i*_)) × 100,000, where r_*i*_ represents age-specific rates and ω_*i*_ represents standard population weights. We analyzed age-standardized prevalence (ASPR), incidence (ASIR), and death rates (ASDR) expressed per 100,000 WCBA (20).

Temporal trends were evaluated using estimated annual percent changes (EAPCs) via log-linear regression: *y* = α + βχ + ε, where y represents ln(ASR), x represents calendar year, and β represents annual change rate. EAPC was calculated as 100 × (eΛβ – 1) with 95% confidence intervals (21). The study period (1990–2021) was analyzed across six intervals: 1990–1994, 1995–1999, 2000–2004, 2005–2009, 2010–2014, and 2015–2021.

### 2.5 Joinpoint regression analysis

We computed the AAPC utilizing the aapc function from R's segmented package via segmented linear regression analysis. This metric represents a weighted mean of annual percentage changes across individual segments (22).

### 2.6 Frontier analysis

To examine the relationship between ICH burden among WCBA and sociodemographic development, a frontier analysis was conducted using a frontier model developed with SDI and age-standardized DALY rate (ASDR). Locally weighted regression (LOESS) and local polynomial regression were applied to create a smoothed boundary line, capturing the nonlinear relationship between SDI and ASDR. The gap between each country's 2021 ASDR and the frontier boundary was measured to estimate potential improvements, with 1,000 bootstrap samples used to ensure analytical robustness (23).

### 2.7 Cross-country inequality analysis

To evaluate disparities in ICH burden among WCBA across countries with varying socioeconomic development levels, we applied two complementary inequality measures following WHO guidelines (24). The Slope Index of Inequality (SII) quantified absolute inequality using: SII = β1, where β1 represents the regression coefficient from DALY_rate = β0 + β1(relative_rank) + ε, with relative_rank representing each country's cumulative population distribution midpoint based on SDI ranking (0–1). The Concentration Index (CI) assessed relative inequality using: CI = (2/n^2^μ) × Σ_*i*_(y_*i*_ × r_*i*_) – 1, where n represents countries, μ is mean DALY rate, *y* represents country DALY rates, and r denotes fractional SDI rank. Both measures were implemented using the ineq package (version 0.2-13) in R. Robust regression modeling employed the MASS package (version 7.3-58) with rlm() function using Huber M-estimation (*k* = 1.345) and convergence criterion of 1 × 10^−4^. LOESS frontier analysis used mgcv package (version 1.8-40) with gam() function and smoothing splines s(SDI, bs = “cs”, *k* = 10). Temporal inequality trends across 204 countries (1990–2021) were smoothed using locally weighted regression (span = 0.3).

### 2.8 Bayesian Age-Period-Cohort model

To further investigate the influence of age, time period, and birth cohort on ICH burden, a Bayesian Age-Period-Cohort (BAPC) model was applied using Bayesian inference to analyze disease burden trends. The model accounts for age effects reflecting biological and physiological factors, period effects capturing environmental and healthcare changes, and cohort effects representing lifetime risk differences among birth cohorts. Markov Chain Monte Carlo (MCMC) method was used for inference, enabling the model to quantify age, period, and cohort effects and forecast future trends (14).

### 2.9 Data analysis and statistical methods

This study assessed ICH burden among WCBA using DALY rates expressed per 100,000 WCBA to measure the disease's impact and case numbers to represent the absolute burden, with both metrics presented with 95% confidence intervals (CIs). All statistical analyses were conducted using R software (version 4.3.1), with Bayesian methods applied for model adjustment using the Meta-Regression Bayesian Regularized Trimmed (MR-BRT) tool to ensure accuracy, and the Wald χ^2^ test used to evaluate annual trends with *p* < 0.05 considered statistically significant.

A detailed flowchart illustrating the complete data extraction, processing, and analytical workflow is provided in the Supplementary Figure S1.

### 2.10 Ethics statement

Under the oversight of the University of Washington's Research Ethics Committee, the GBD study strictly follows its licensing agreement (https://www.healthdata.org/research-analysis/gbd). Since this study relies on publicly available data, all data collection and usage comply with the Ethics Committee's regulations and the Guidelines for Accurate and Transparent Health Estimates Reporting (GATHER). As a result, no additional ethical review is required.

### 2.11 Funding transparency

The study sponsors exercised no influence over research design, data gathering and evaluation, result interpretation, or manuscript development. Complete data access remains with the corresponding author, who bears final responsibility for publication submission.

## 3 Results

### 3.1 Global burden trends

#### 3.1.1 Incidence

**Figure 1:**
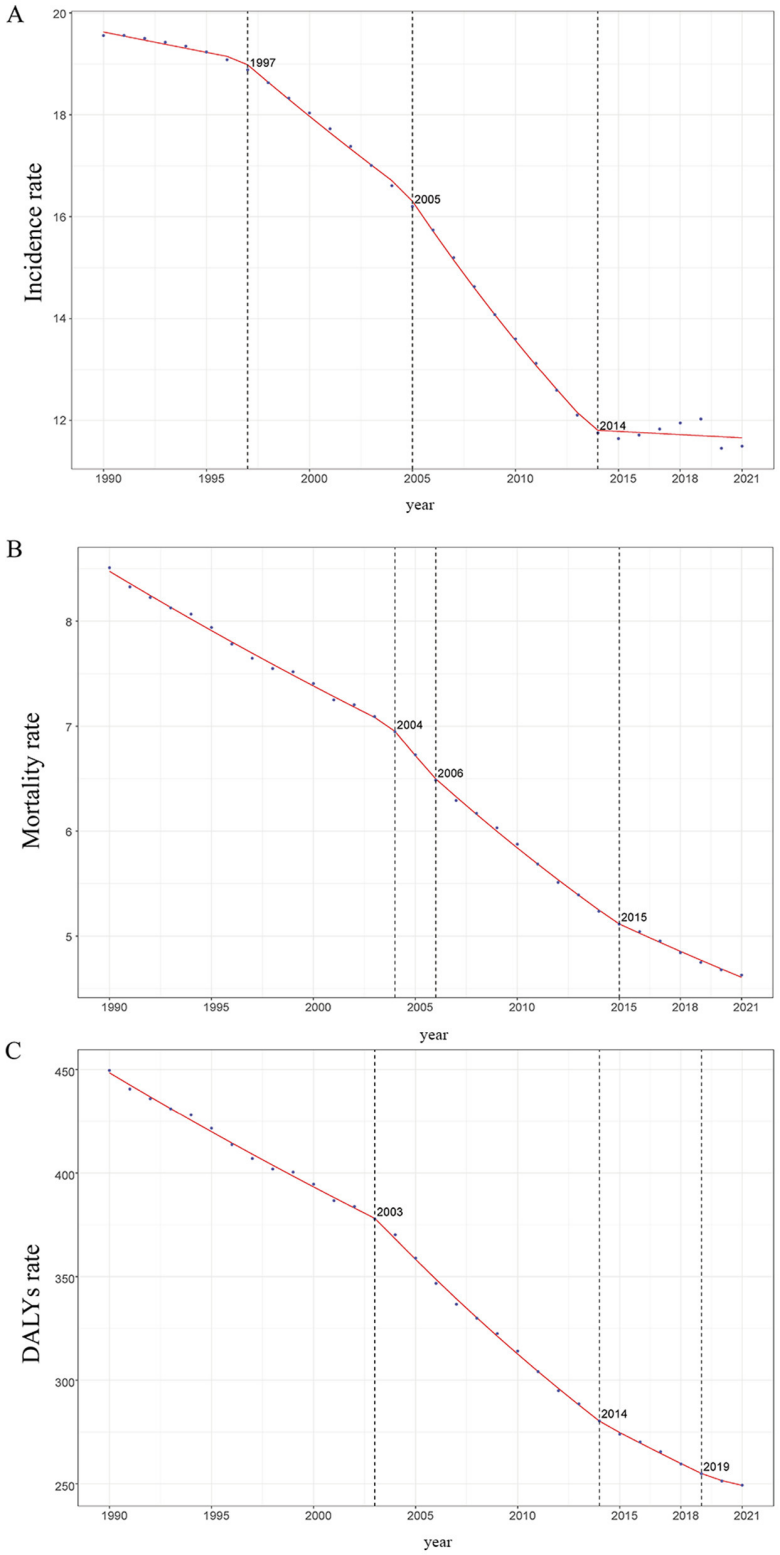
Trends in global incidence, mortality, and disability - adjusted life years (DALYs) of ICH among WCBA from 1990 to 2021, as Illustrated by APCC Data Plots. **(A)** Incidence rate. **(B)** Mortality rate. **(C)** DALYs rate.

Large-scale data analysis reveals a declining trend in the global incidence of ICH among WCBA. Between 1990 and 2021, the incidence rate exhibited a downward trajectory. Initially, in 1990, the incidence rate was relatively high, followed by a gradual decline from 1990 to 1997. This downward trend continued from 1997 to 2005, with an accelerated rate of decline. Between 2005 and 2014, the incidence rate kept decreasing, but at a progressively slower pace. From 2014 to 2021, the incidence rate stabilized at a relatively low level, showing only minor fluctuations (Figure 1A).

**Figure 2:**
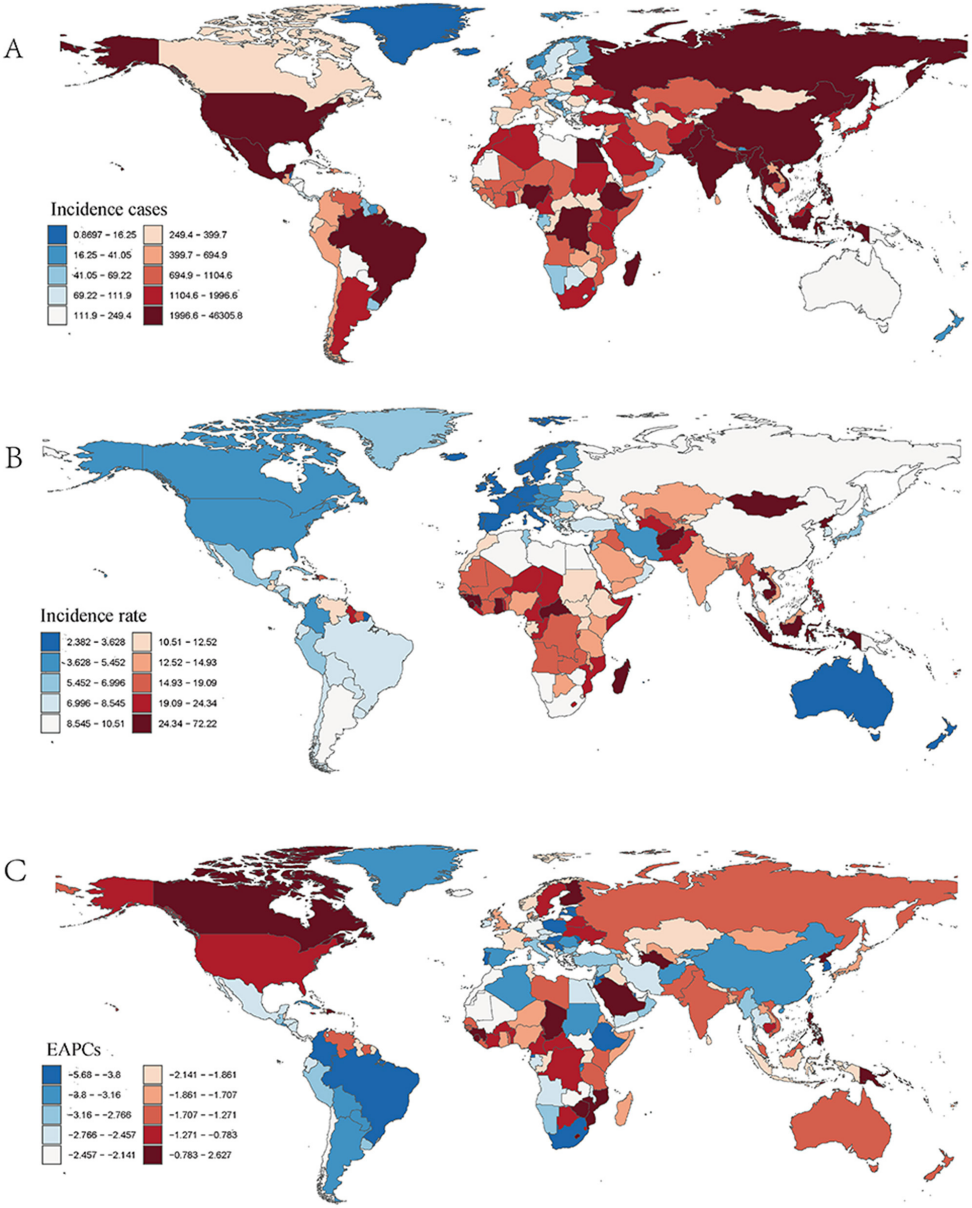
Incidence of ICH among WCBA across 204 countries and territories. **(A)** Number of incidence cases. **(B)** Incidence rate. **(C)** Estimated annual percentage change (EAPC) in incidence.

The estimated annual percentage change (EAPC) from 1990 to 2021 was −2.106% (95% CI: −2.284% to −1.927%; Table 1; Figure 2C). In 1990, the global incidence of ICH among WCBA was estimated at 232,483.472 cases (95% uncertainty interval: 159,709.013–325,715.331), with an incidence rate of 19.556 per 100,000 (95% UI: 13.508–27.257). By 2021, the number of cases slightly declined to 231,888.269 (95% UI: 163,210.539–320,866.825), while the incidence rate significantly dropped to 11.493 per 100,000 (95% UI: 8.073–15.925), reflecting an overall reduction of 41.23% (Table 1; Figure 2).

**Table 1:** Incidence of Intracerebral Hemorrhage among women of childbearing age between 1990 and 2021 at the global and regional level.

**Location**	**Rate per 100,000 (95% UI)**						
	**1990**		**2021**		**1990–2021**		
	**Incident cases**	**Incident rate**	**Incident cases**	**Incident rate**	**Cases change^b^**	**Rate change^b^**	**EAPCs^a^**
Global	232,483.472 (159,709.013, 325,715.331)	19.556 (13.508, 27.257)	231,888.269 (163,210.539, 320,866.825)	11.493 (8.073, 15.925)	−0.26 (163,210.539, 320,866.825)	−41.23 (8.073, 15.925)	−2.106 (−2.284, −1.927)
**SDI**							
Low SDI	25,038.734 (18,188.586, 34,042.133)	28.098 (20.578, 37.956)	36,736.937 (27,045.522, 48,805.629)	16.423 (12.217, 21.640)	46.72 (27,045.522, 48,805.629)	−41.55 (12.217, 21.640)	−2.035 (−2.158, −1.911)
Low–middle SDI	53,244.361 (37,010.060, 73,675.499)	23.348 (16.331, 32.068)	73,182.977 (52,146.757, 100,188.177)	15.447 (11.042, 21.109)	37.45 (52,146.757, 100,188.177)	−33.84 (11.042, 21.109)	−1.557 (−1.684, −1.430)
Middle SDI	85,245.644 (57,105.974, 121,574.458)	22.832 (15.394, 32.338)	77,220.655 (53,261.143, 108,676.111)	11.600 (7.970, 16.375)	−9.41 (53,261.143, 108,676.111)	−49.19 (7.970, 16.375)	−2.612 (−2.805, −2.418)
High–middle SDI	48,866.156 (33,340.108, 69,062.761)	19.105 (13.093, 26.914)	31,573.083 (21,532.515, 44,912.533)	8.898 (5.980, 12.798)	−35.39 (21,532.515, 44,912.533)	−53.43 (5.980, 12.798)	−3.050 (−3.366, −2.733)
High SDI	19,877.886 (13,357.796, 28,570.983)	8.540 (5.708, 12.336)	12,978.864 (8,434.783, 19,198.380)	4.816 (3.057, 7.250)	−34.71 (8,434.783, 19,198.380)	−43.61 (3.057, 7.250)	−2.197 (−2.347, −2.046)
**Regions**							
East Asia	69,195.371 (45,098.159, 100,344.244)	24.494 (16.053, 35.304)	40,932.614 (27,510.336, 58,870.682)	10.311 (6.869, 14.931)	−40.84 (27,510.336, 58,870.682)	−57.9 (6.869, 14.931)	−3.409 (−3.727, −3.090)
Southeast Asia	31,170.498 (21,706.919, 43,119.711)	31.356 (22.022, 43.074)	38,783.697 (27,894.634, 52,740.130)	20.186 (14.482, 27.490)	24.42 (27,894.634, 52,740.130)	−35.62 (14.482, 27.490)	−1.633 (−1.804, −1.462)
Oceania	280.729 (205.840, 374.274)	22.146 (16.441, 29.196)	522.675 (392.423, 691.194)	16.363 (12.341, 21.558)	86.18 (392.423, 691.194)	−26.11 (12.341, 21.558)	−1.050 (−1.187, −0.913)
Central Asia	3,434.670 (2,695.866, 4,367.931)	25.680 (20.557, 32.154)	3,744.564 (2,919.353, 4,778.592)	15.064 (11.713, 19.290)	9.02 (2,919.353, 4,778.592)	−41.34 (11.713, 19.290)	−1.954 (−2.220, −1.687)
Central Europe	4,284.385 (3,273.630, 5,574.525)	13.470 (10.250, 17.618)	1,586.420 (1,126.361, 2,207.265)	5.340 (3.638, 7.689)	−62.97 (1,126.361, 2,207.265)	−60.36 (3.638, 7.689)	−3.486 (−3.704, −3.268)
Eastern Europe	7,499.655 (4,945.366, 10,877.987)	13.585 (8.952, 19.759)	5,667.416 (3,872.408, 7,954.977)	9.521 (6.419, 13.538)	−24.43 (3,872.408, 7,954.977)	−29.92 (6.419, 13.538)	−1.448 (−1.862, −1.032)
High-income Asia Pacific	6,979.886 (4,790.948, 9,853.709)	14.390 (9.823, 20.410)	2,845.908 (1,822.166, 4,276.182)	6.251 (3.832, 9.700)	−59.23 (1,822.166, 4,276.182)	−56.56 (3.832, 9.700)	−3.318 (−3.564, −3.071)
Australasia	219.636 (134.036, 337.224)	4.050 (2.464, 6.239)	196.290 (108.118, 315.097)	2.542 (1.372, 4.146)	−10.63 (108.118, 315.097)	−37.23 (1.372, 4.146)	−1.604 (−1.786, −1.422)
Western Europe	6,013.499 (4,037.251, 8,651.168)	6.084 (4.054, 8.809)	3,252.784 (1,932.678, 5,099.170)	3.142 (1.802, 5.049)	−45.91 (1,932.678, 5,099.170)	−48.36 (1.802, 5.049)	−2.518 (−2.676, −2.360)
Southern Latin America	2,525.038 (1,864.683, 3,339.563)	21.028 (15.579, 27.740)	1,616.655 (1,128.459, 2,243.423)	8.911 (6.168, 12.442)	−35.98 (1,128.459, 2,243.423)	−57.62 (6.168, 12.442)	−3.122 (−3.340, −2.905)
High-income North America	4,045.661 (2,363.200, 6,348.608)	5.316 (3.080, 8.398)	3,493.746 (2,120.145, 5,374.415)	3.920 (2.345, 6.092)	−13.64 (2,120.145, 5,374.415)	−26.26 (2.345, 6.092)	−1.019 (−1.103, −0.935)
Caribbean	1,734.222 (1,355.526, 2,220.367)	21.429 (16.940, 27.187)	1,942.919 (1,525.934, 2,479.234)	15.782 (12.374, 20.157)	12.03 (1,525.934, 2,479.234)	−26.35 (12.374, 20.157)	−1.245 (−1.377, −1.113)
Andean Latin America	1,373.100 (1,023.908, 1,824.319)	16.506 (12.458, 21.755)	1,196.535 (829.583, 1,674.703)	6.900 (4.785, 9.661)	−12.86 (829.583, 1,674.703)	−58.2 (4.785, 9.661)	−3.089 (−3.236, −2.941)
Central Latin America	4,947.175 (3,496.329, 6,848.740)	13.925 (10.044, 18.983)	4,557.622 (3,077.135, 6,499.890)	6.589 (4.438, 9.415)	−7.87 (3,077.135, 6,499.890)	−52.68 (4.438, 9.415)	−2.780 (−2.965, −2.595)
Tropical Latin America	8,621.547 (5,635.756, 12,552.457)	25.139 (16.502, 36.444)	5,153.000 (3,419.254, 7,425.103)	7.909 (5.215, 11.464)	−40.23 (3,419.254, 7,425.103)	−68.54 (5.215, 11.464)	−4.330 (−4.588, −4.070)
North Africa and Middle East	12,939.376 (9,570.959, 17,289.284)	19.639 (14.719, 25.954)	15,621.492 (11,664.559, 20,702.268)	9.785 (7.301, 12.983)	20.73 (11,664.559, 20,702.268)	−50.18 (7.301, 12.983)	−2.583 (−2.718, −2.448)
South Asia	42,798.782 (28,024.137, 62,381.292)	19.673 (12.954, 28.516)	64,452.171 (43,753.453, 92,002.347)	13.765 (9.365, 19.628)	50.59 (43,753.453, 92,002.347)	−30.03 (9.365, 19.628)	−1.334 (−1.427, −1.241)
Central Sub-Saharan Africa	2,501.716 (1,827.203, 3,372.330)	26.335 (19.421, 35.154)	4,578.850 (3,400.897, 6,062.959)	17.464 (13.114, 22.881)	83.03 (3,400.897, 6,062.959)	−33.69 (13.114, 22.881)	−1.503 (−1.629, −1.376)
Eastern Sub-Saharan Africa	11,106.955 (8,129.325, 14,942.794)	34.286 (25.302, 45.798)	13,299.626 (9,900.738, 17,602.566)	15.746 (11.885, 20.648)	19.74 (9,900.738, 17,602.566)	−54.07 (11.885, 20.648)	−2.968 (−3.140, −2.794)
Southern Sub-Saharan Africa	2,301.287 (1,529.813, 3,294.734)	21.148 (14.153, 29.985)	1,908.192 (1,308.311, 2,676.116)	9.119 (6.264, 12.790)	−17.08 (1,308.311, 2,676.116)	−56.88 (6.264, 12.790)	−3.233 (−3.567, −2.898)
Western Sub-Saharan Africa	8,510.285 (6,171.811, 11,484.839)	25.730 (18.869, 34.401)	16,535.093 (12,115.103, 22,226.831)	17.100 (12.664, 22.800)	94.3 (12,115.103, 22,226.831)	−33.54 (12.664, 22.800)	−1.537 (−1.691, −1.383)

EAPC, estimated annual percentage change; SDI, Sociodemographic Index; UI, uncertainty interval.

^a^EAPC is expressed as 95% confidence interval.

^b^Change shows the percentage change.

**Figure 3:**
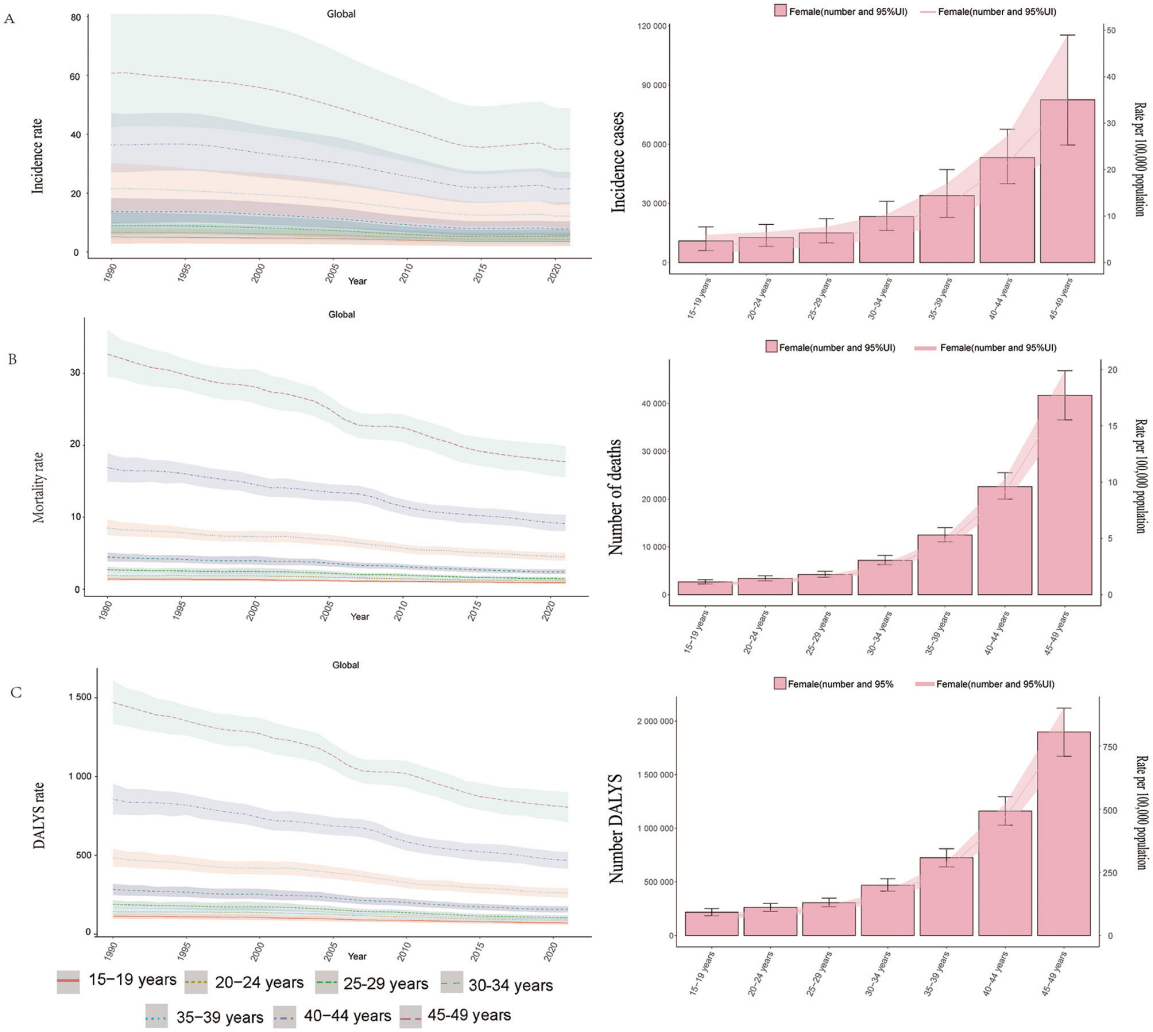
Trends in incidence, mortality, and disability - adjusted life yeas (DALYs) of ICH among WCBA by age, 1990–2021. **(A)** Incidence rate. **(B)** Mortality rate. **(C)** DALYs rate.

When analyzing different age groups (Figure 3A), notable variations in incidence were observed. Older age groups within the WCBA range had higher incidence rates. In both 1990 and 2021, the 45–49 age group exhibited the highest incidence, whereas the 15–19 age group had the lowest incidence, contributing minimally to the total cases. In 2021, older age groups accounted for a larger share of the total incidence burden (Figure 3A; Supplementary Figure S4A).

#### 3.1.2 Mortality

Similar to incidence trends, the mortality rate of ICH among WCBA has consistently declined over the past three decades. Between 1990 and 2004, the decline was gradual. However, from 2004 to 2008, the rate of decline accelerated, and from 2008 to 2015, it further intensified. In 2015–2021, the mortality rate continued to drop, albeit with some fluctuations (Figure 1B).

Between 1990 and 2021, the EAPC for mortality was −2.081% (95% CI: −2.180% to −1.982%; Table 2; Supplementary Figure S2C). In 1990, the global number of deaths from ICH among WCBA was 98,138.675 (95% UI: 86,772.757–110,210.702), with a mortality rate of 8.510 per 100,000 (95% UI: 7.545–9.531). By 2021, deaths had decreased to 94,223.648 (95% UI: 82,658.556–106,577.710), and the mortality rate dropped significantly to 4.628 per 100,000 (95% UI: 4.059–5.237), representing an overall decrease of 45.62%.

**Table 2:** Mortality of Intracerebral Hemorrhage among women of childbearing age between 1990 and 2021 at the global and regional level.

**Location**	**Rate per 100,000 (95% UI)**						
	**1990**		**2021**		**1990–2021**		
	**Number of deaths**	**Mortality rate**	**Number of deaths**	**Mortality rate**	**Cases change^b^**	**Rate change^b^**	**EAPC^a^**
Global	98,138.675 (86,772.757, 110,210.702)	8.510 (7.545, 9.531)	94,223.648 (82,658.556, 106,577.710)	4.628 (4.059, 5.237)	−3.99 (82,658.556, 106,577.710)	−45.62 (4.059, 5.237)	−2.081 (−2.180, −1.982)
**SDI**							
Low SDI	10,975.910 (8,342.888, 14,043.051)	12.572 (9.627, 16.018)	16,176.516 (12,482.730, 19,854.715)	7.352 (5.692, 9.002)	47.38 (12,482.730, 19,854.715)	−41.52 (5.692, 9.002)	−1.955 (−2.048, −1.862)
Low–middle SDI	25,034.736 (20,401.676, 30,133.747)	11.239 (9.220, 13.433)	30,355.254 (25,226.233, 35,949.700)	6.485 (5.392, 7.674)	21.25 (25,226.233, 35,949.700)	−42.30 (5.392, 7.674)	−1.778 (−1.865, −1.692)
Middle SDI	37,568.145 (33,182.365, 42,915.770)	10.581 (9.348, 12.070)	32,973.024 (28,587.774, 37,786.555)	4.838 (4.194, 5.547)	−12.23 (28,587.774, 37,786.555)	−54.28 (4.194, 5.547)	−2.581 (−2.664, −2.499)
High–middle SDI	18,588.927 (16,053.719, 21,587.147)	7.513 (6.496, 8.713)	11,397.507 (9,723.868, 13,333.055)	2.990 (2.550, 3.498)	−38.69 (9,723.868, 13,333.055)	−60.20 (2.550, 3.498)	−3.282 (−3.629, −2.935)
High SDI	5,862.601 (5,478.467, 6,277.690)	2.496 (2.332, 2.674)	3,217.700 (2,885.041, 3,622.031)	1.110 (0.993, 1.253)	−45.11 (2,885.041, 3,622.031)	−55.53 (0.993, 1.253)	−2.757 (−2.875, −2.638)
**Regions**							
East Asia	28,557.973 (22,465.357, 36,112.398)	10.702 (8.430, 13.483)	16,449.806 (12,483.084, 21,001.124)	3.885 (2.950, 4.971)	−42.40 (12,483.084, 21,001.124)	−63.70 (2.950, 4.971)	−3.336 (−3.607, −3.064)
Southeast Asia	17,479.107 (14,670.126, 20,673.872)	18.228 (15.426, 21.375)	21,347.742 (17,392.358, 26,719.333)	10.989 (8.954, 13.761)	22.13 (17,392.358, 26,719.333)	−39.71 (8.954, 13.761)	−1.613 (−1.713, −1.512)
Oceania	257.449 (155.192, 406.497)	21.307 (13.009, 33.197)	503.859 (319.691, 752.619)	15.997 (10.231, 23.761)	95.71 (319.691, 752.619)	−24.92 (10.231, 23.761)	−0.982 (−1.070, −0.893)
Central Asia	1,240.986 (1,131.126, 1,356.758)	9.685 (8.841, 10.573)	933.412 (782.344, 1,116.704)	3.749 (3.143, 4.483)	−24.78 (782.344, 1,116.704)	−61.29 (3.143, 4.483)	−3.726 (−4.136, −3.314)
Central Europe	1,812.510 (1,696.946, 1,933.197)	5.658 (5.296, 6.037)	464.740 (404.630, 530.983)	1.374 (1.196, 1.571)	−74.36 (404.630, 530.983)	−75.72 (1.196, 1.571)	−5.083 (−5.397, −4.769)
Eastern Europe	2,471.494 (2,296.565, 2,618.627)	4.531 (4.211, 4.801)	2,038.400 (1,757.600, 2,353.268)	3.229 (2.785, 3.730)	−17.52 (1,757.600, 2,353.268)	−28.74 (2.785, 3.730)	−1.926 (−2.494, −1.355)
High-income Asia Pacific	1,647.677 (1,413.090, 1,894.726)	3.306 (2.825, 3.816)	472.140 (424.984, 534.167)	0.905 (0.811, 1.032)	−71.35 (424.984, 534.167)	−72.63 (0.811, 1.032)	−4.391 (−4.687, −4.094)
Australasia	49.774 (41.572, 59.190)	0.920 (0.768, 1.093)	31.250 (25.105, 38.025)	0.374 (0.301, 0.456)	−37.22 (25.105, 38.025)	−59.35 (0.301, 0.456)	−2.836 (−3.037, −2.634)
Western Europe	1,928.995 (1,813.145, 2,057.883)	1.929 (1.814, 2.058)	563.307 (526.117, 602.069)	0.494 (0.462, 0.528)	−70.80 (526.117, 602.069)	−74.39 (0.462, 0.528)	−4.580 (−4.702, −4.458)
Southern Latin America	832.269 (724.952, 943.783)	7.006 (6.106, 7.941)	334.087 (281.878, 387.068)	1.787 (1.506, 2.072)	−59.86 (281.878, 387.068)	−74.49 (1.506, 2.072)	−4.439 (−4.641, −4.236)
High-income North America	1192.755 (1134.676, 1254.227)	1.559 (1.484, 1.639)	1018.505 (953.875, 1085.452)	1.083 (1.014, 1.155)	−14.61 (953.875, 1085.452)	−30.53 (1.014, 1.155)	−1.246 (−1.437, −1.054)
Caribbean	855.137 (671.106, 1,078.330)	10.751 (8.500, 13.453)	941.606 (658.632, 1,328.518)	7.617 (5.319, 10.771)	10.11 (658.632, 1,328.518)	−29.15 (5.319, 10.771)	−1.097 (−1.285, −0.908)
Central Latin America	1,688.795 (1,580.516, 1,796.640)	5.067 (4.748, 5.387)	1,487.798 (1,232.307, 1,775.931)	2.131 (1.765, 2.544)	−11.90 (1,232.307, 1,775.931)	−57.94 (1.765, 2.544)	−3.326 (−3.683, −2.968)
Tropical Latin America	3,840.190 (3,637.879, 4,054.996)	11.636 (11.034, 12.276)	2,270.644 (2,113.895, 2,440.048)	3.416 (3.179, 3.671)	−40.87 (2,113.895, 2,440.048)	−70.64 (3.179, 3.671)	−4.414 (−4.561, −4.266)
North Africa and Middle East	6,584.051 (5,175.366, 8,006.510)	10.412 (8.197, 12.613)	7,234.072 (5,504.834, 9,295.584)	4.544 (3.458, 5.840)	9.87 (5,504.834, 9,295.584)	−56.36 (3.458, 5.840)	−2.786 (−2.910, −2.661)
South Asia	16,291.037 (11,946.640, 20,891.176)	7.701 (5.708, 9.789)	21,854.911 (16,800.447, 27,041.665)	4.715 (3.628, 5.829)	34.15 (16,800.447, 27,041.665)	−38.77 (3.628, 5.829)	−1.555 (−1.628, −1.483)
Central Sub-Saharan Africa	1,062.357 (662.773, 1,590.903)	11.399 (7.144, 17.017)	1,930.039 (1,204.935, 2,965.674)	7.520 (4.722, 11.548)	81.68 (1,204.935, 2,965.674)	−34.03 (4.722, 11.548)	−1.473 (−1.567, −1.378)
Eastern Sub-Saharan Africa	4,650.449 (3,481.569, 6,300.489)	14.608 (11.014, 19.810)	6,050.460 (4,732.554, 7,604.030)	7.289 (5.732, 9.127)	30.10 (4,732.554, 7,604.030)	−50.10 (5.732, 9.127)	−2.608 (−2.783, −2.433)
Southern Sub-Saharan Africa	1,176.324 (995.540, 1,376.698)	11.139 (9.445, 13.004)	1,351.090 (1,083.233, 1,670.557)	6.601 (5.296, 8.152)	14.86 (1,083.233, 1,670.557)	−40.74 (5.296, 8.152)	−0.807 (−1.537, −0.072)
Western Sub-Saharan Africa	3,877.895 (2,953.122, 5,057.433)	12.157 (9.316, 15.803)	6,478.457 (4,623.106, 8,390.341)	6.876 (4.915, 8.905)	67.06 (4,623.106, 8,390.341)	−43.44 (4.915, 8.905)	−1.886 (−1.972, −1.799)

EAPC, estimated annual percentage change; SDI, Sociodemographic Index; UI, uncertainty interval.

^a^EAPC is expressed as 95% confidence interval.

^b^Change shows the percentage change.

Age-based variations in mortality rates were evident. Older age groups, particularly the 45–49 age group, had higher mortality rates, contributing significantly to total deaths. Conversely, the 15–19 age group had lower mortality rates, with a smaller impact on overall deaths (Figure 3B, Supplementary Figure S4B).

#### 3.1.3 DALYs

Following the trends in incidence and mortality, the disability-adjusted life years (DALYs) rate associated with ICH among WCBA has shown a consistent decline over the past three decades. Between 1990 and 2003, the DALYs rate declined gradually. From 2003 to 2014, the rate of decline accelerated, as reflected by a steeper curve. Between 2014 and 2019, the decline slowed again, and from 2019 to 2021, the DALYs rate continued to decrease, though with some fluctuations (Figure 1C).

From 1990 to 2021, the EAPC for DALYs was −2.034% (95% CI: −2.135% to −1.933%; Table 1; Supplementary Figure S3C). In 1990, global DALYs for ICH among WCBA were 5,311,065.424 (95% UI: 4,707,132.784–5,929,971.703), with a DALYs rate of 449.575 per 100,000 (95% UI: 399.478–500.814). By 2021, DALYs had dropped to 5,040,596.199 (95% UI: 4,432,533.780–5,654,101.616), with a DALYs rate of 249.334 per 100,000 (95% UI: 219.195–279.770), representing a total decrease of 44.54% (Table 3; Supplementary Figure S3).

**Table 3:** Disability-adjusted life years (DALYs) of Intracerebral Hemorrhage among women of childbearing age between 1990 and 2021 at the global and regional level.

**Location**	**Rate per 100,000 (95% UI)**						
	**1990**		**2021**		**1990–2021**		
	**Number of DALYs**	**DALYs rate**	**Number of DALYs**	**DALYs rate**	**Cases change^b^**	**Rate change^b^**	**EAPC^a^**
Global	5,311,065.424 (4,707,132.784, 5,929,971.703)	449.575 (399.478, 500.814)	5,040,596.199 (4,432,533.780, 5,654,101.616)	249.334 (219.195, 279.770)	−5.09 (4,432,533.780, 5,654,101.616)	−44.54 (219.195, 279.770)	−2.034 (−2.135, −1.933)
**SDI**							
Low SDI	585,897.418 (448,919.716, 745,919.294)	646.728 (499.358, 820.158)	874,107.394 (681,114.854, 1,068,068.774)	383.105 (299.163, 466.822)	49.19 (681,114.854, 1,068,068.774)	−40.76 (299.163, 466.822)	−1.912 (−2.004, −1.821)
Low–middle SDI	1,338,855.046 (1,095,239.326, 1,611,662.833)	583.248 (480.133, 696.840)	1,602,343.824 (1,342,230.719, 1,877,152.884)	338.443 (283.639, 396.174)	19.68 (1,342,230.719, 1,877,152.884)	−41.97 (283.639, 396.174)	−1.788 (−1.878, −1.697)
Middle SDI	2,029,073.299 (1,794,818.459, 2,306,004.207)	553.025 (489.380, 627.672)	1,740,808.252 (1,522,353.963, 1,976,768.342)	258.882 (226.435, 294.069)	−14.21 (1,522,353.963, 1,976,768.342)	−53.19 (226.435, 294.069)	−2.527 (−2.615, −2.439)
High–middle SDI	1,013,669.024 (880,130.107, 1,167,572.375)	401.238 (348.646, 461.692)	621,062.203 (536,058.562, 716,899.653)	168.708 (145.652, 194.757)	−38.73 (536,058.562, 716,899.653)	−57.95 (145.652, 194.757)	−3.103 (−3.418, −2.787)
High SDI	337,807.304 (312,157.364, 365,526.772)	144.349 (133.309, 156.279)	196,821.703 (175,909.227, 221,142.106)	70.510 (62.742, 79.522)	−41.74 (175,909.227, 221,142.106)	−51.15 (62.742, 79.522)	−2.437 (−2.534, −2.341)
**Regions**							
East Asia	1,547,030.028 (1,243,198.134, 1,924,843.855)	561.473 (450.949, 696.857)	886,797.338 (695,516.580, 1,106,994.182)	217.645 (170.717, 271.894)	−42.68 (695,516.580, 1,106,994.182)	−61.24 (170.717, 271.894)	−3.161 (−3.414, −2.907)
Southeast Asia	938,111.123 (788,569.094, 1,113,341.721)	947.309 (803.494, 1,114.071)	1,102,937.651 (910,580.089, 1,358,801.090)	571.842 (472.226, 704.777)	17.57 (910,580.089, 1,358,801.090)	−39.64 (472.226, 704.777)	−1.633 (−1.742, −1.525)
Oceania	13,399.853 (8,171.886, 21,155.392)	1,074.609 (663.344, 1,673.015)	26,049.751 (16,731.476, 38,835.904)	815.204 (527.422, 1,208.445)	94.40 (16,731.476, 38,835.904)	−24.14 (527.422, 1,208.445)	−0.957 (−1.052, −0.861)
Central Asia	68,601.260 (62,421.167, 75,103.437)	511.683 (466.504, 559.322)	51,579.171 (43,750.574, 60,971.872)	207.252 (175.841, 244.973)	−24.81 (43,750.574, 60,971.872)	−59.50 (175.841, 244.973)	−3.573 (−3.948, −3.197)
Central Europe	96,727.490 (90,141.676, 103,742.090)	302.672 (281.893, 324.885)	27,052.715 (23,711.433, 30,841.063)	84.795 (74.053, 96.893)	−72.03 (23,711.433, 30,841.063)	−71.98 (74.053, 96.893)	−4.625 (−4.892, −4.358)
Eastern Europe	130,417.831 (121,040.515, 139,300.330)	237.342 (220.341, 253.388)	106,855.268 (92,680.784, 122,328.415)	174.025 (150.923, 199.341)	−18.07 (92,680.784, 122,328.415)	−26.68 (150.923, 199.341)	−1.769 (−2.300, −1.235)
High-income Asia Pacific	95,734.272 (82,229.913, 110,179.457)	195.756 (167.573, 226.068)	31,506.456 (27,535.919, 36,332.685)	66.169 (57.015, 77.230)	−67.09 (27,535.919, 36,332.685)	−66.20 (57.015, 77.230)	−3.724 (−3.979, −3.468)
Australasia	3,036.786 (2,574.406, 3,585.793)	55.987 (47.491, 66.083)	2,138.834 (1,746.073, 2,600.035)	26.611 (21.662, 32.407)	−29.57 (1,746.073, 2,600.035)	−52.47 (21.662, 32.407)	−2.447 (−2.596, −2.299)
Western Europe	109,276.871 (102,092.398, 117,087.811)	110.195 (102.920, 118.106)	36,723.908 (33,094.625, 40,877.546)	34.015 (30.465, 38.094)	−66.39 (33,094.625, 40,877.546)	−69.13 (30.465, 38.094)	−4.017 (−4.139, −3.895)
Southern Latin America	45,691.568 (39,900.060, 51,686.231)	381.982 (333.744, 431.885)	20,299.503 (17,179.929, 23,593.491)	110.254 (93.225, 128.297)	−55.57 (17,179.929, 23,593.491)	−71.14 (93.225, 128.297)	−4.059 (−4.269, −3.849)
High-income North America	71,328.958 (65,969.713, 77,269.928)	93.168 (86.134, 100.969)	62,301.551 (56,926.446, 68,274.376)	67.909 (61.842, 74.702)	−12.66 (56,926.446, 68,274.376)	−27.11 (61.842, 74.702)	−1.045 (−1.205, −0.884)
Caribbean	45,024.684 (35,238.155, 56,963.620)	552.611 (435.845, 693.278)	49,027.056 (34,396.038, 69,185.732)	398.641 (279.212, 563.862)	8.89 (34,396.038, 69,185.732)	−27.86 (279.212, 563.862)	−1.011 (−1.190, −0.832)
Andean Latin America	35,825.667 (27,821.699, 46,688.022)	429.994 (335.673, 557.426)	25,854.598 (18,745.460, 35,043.052)	149.338 (108.270, 202.379)	−27.83 (18,745.460, 35,043.052)	−65.27 (108.270, 202.379)	−3.847 (−4.105, −3.589)
Central Latin America	94,050.621 (87,906.071, 100,242.452)	270.310 (252.945, 287.839)	81,237.880 (68,401.102, 95,997.531)	116.882 (98.417, 138.102)	−13.62 (68,401.102, 95,997.531)	−56.76 (98.417, 138.102)	−3.201 (−3.543, −2.857)
Tropical Latin America	197,523.021 (187,098.499, 208,849.200)	584.872 (554.697, 617.892)	116,055.836 (108,063.541, 124,441.969)	176.348 (164.143, 189.126)	−41.24 (108,063.541, 124,441.969)	−69.85 (164.143, 189.126)	−4.323 (−4.475, −4.170)
North Africa and Middle East	366,010.078 (291,079.696, 442,685.750)	555.447 (442.077, 668.981)	394,696.838 (306,965.683, 500,927.767)	247.472 (192.468, 314.172)	7.84 (306,965.683, 500,927.767)	−55.45 (192.468, 314.172)	−2.726 (−2.840, −2.612)
South Asia	873,208.316 (648,240.369, 1,114,685.540)	401.239 (301.273, 507.467)	1,162,960.151 (907,217.040, 1,424,833.793)	248.437 (193.899, 304.214)	33.18 (907,217.040, 1,424,833.793)	−38.08 (193.899, 304.214)	−1.548 (−1.612, −1.485)
Central Sub-Saharan Africa	56,238.861 (35,849.147, 83,153.820)	579.332 (370.721, 854.861)	101,876.203 (64,961.162, 154,221.588)	383.328 (245.851, 580.260)	81.15 (64,961.162, 154,221.588)	−33.83 (245.851, 580.260)	−1.462 (−1.557, −1.366)
Eastern Sub-Saharan Africa	250,802.243 (188,900.079, 335,693.651)	751.802 (570.545, 1,008.037)	327,673.219 (257,728.806, 409,307.575)	378.183 (299.101, 470.382)	30.65 (257,728.806, 409,307.575)	−49.70 (299.101, 470.382)	−2.582 (−2.749, −2.414)
Southern Sub-Saharan Africa	63,942.762 (54,395.708, 74,589.727)	585.258 (498.492, 680.778)	69,479.114 (56,034.979, 85,507.387)	335.751 (270.908, 412.787)	8.66 (56,034.979, 85,507.387)	−42.63 (270.908, 412.787)	−1.028 (−1.782, −0.269)
Western Sub-Saharan Africa	209,083.130 (160,679.648, 270,786.588)	626.120 (483.966, 808.586)	357,493.156 (261,011.753, 455,904.532)	363.926 (266.114, 464.660)	70.98 (261,011.753, 455,904.532)	−41.88 (266.114, 464.660)	−1.795 (−1.883, −1.708)

EAPC, estimated annual percentage change; SDI, Sociodemographic Index; UI, uncertainty interval.

^a^EAPC is expressed as 95% confidence interval.

^b^Change shows the percentage change.

Age-group analysis revealed that older WCBA age groups (especially 45–49 years old) had higher DALYs rates, while younger age groups (15–19 years old) had lower rates, contributing less to the overall DALYs burden (Figure 3C, Supplementary Figure S4C).

### 3.2 SDI regional analysis

Compared to 1990, by 2021, low SDI regions experienced an increase in incidence, mortality, and DALYs. Incidence cases increased from 25,038.734 (95% UI: 18,188.586–34,042.133) in 1990 to 36,736.937 (95% UI: 27,045.522–48,805.629) in 2021, a 46.72% increase. Mortality cases rose from 10,975.910 (95% UI: 8,342.888–14,043.051) to 16,176.516 (95% UI: 12,482.730–19,854.715), an increase of 47.38%. DALYs increased from 585,897.418 (95% UI: 448,919.716–745,919.294) to 874,107.394 (95% UI: 681,114.854–1,068,068.774), a 49.19% rise (Tables 1–3; Supplementary Figure S7).

In contrast, high SDI and high-middle SDI regions showed a decline across all indicators: High-middle SDI regions: Incidence dropped from 48,866.156 to 31,573.083 (−35.39%). High SDI regions: Incidence decreased from 19,877.886 to 12,978.864 (−34.71%).

Further analysis revealed that EAPC varied significantly by SDI category: Incidence EAPC was highest in low-middle SDI regions (−1.557%; 95% CI: −1.684 to −1.430). Mortality EAPC was highest in low SDI regions (−1.955%; 95% CI: −2.048 to −1.862). DALYs EAPC was highest in low SDI regions (−1.912%; 95% CI: −2.004 to −1.821).

In 2021, the burden of ICH among WCBA was inversely correlated with SDI. Certain high-income regions, such as Western Europe and North America, had a higher-than-expected burden, while regions like sub-Saharan East Africa and Andean Latin America exhibited lower-than-expected burdens (Supplementary Figures S5, S6). Countries such as Switzerland, France, and Kenya had higher-than-expected burdens, while Argentina, the Philippines, and Pakistan had lower-than-expected burdens.

### 3.3 National trends

#### 3.3.1 Incidence

In 2021, India recorded the highest number of ICH cases among WCBA globally, with an estimated 46,305.798 cases [95% uncertainty interval (UI): 30,778.656–67,070.392]. In contrast, the Solomon Islands had the highest incidence rate, at 72.220 cases per 100,000 population (95% UI: 56.566–90.468; Supplementary Table S1; Figures 2A, B). Between 1990 and 2021, the Philippines experienced the largest increase in incidence rate, with an estimated annual percentage change (EAPC) of 2.627% (95% CI: 2.038–3.221%), while the Maldives had the most significant decline, with an EAPC of −5.680% (95% CI: −6.025 to −5.335%; Supplementary Table S1; Figure 2C). In 2021, the global incidence rate of ICH among WCBA was 11.493 per 100,000 population (95% UI: 8.073–15.925), which was higher than that of 180 countries, but lower than 24 countries worldwide (Table 2; Supplementary Table S1). Incidence rates and case numbers varied significantly by region. In Asia, China's case numbers declined from 66,558.020 (95% UI: 43,007.008–96,945.680) in 1990 to 38,547.567 in 2021, with the incidence rate falling from 24.454 to 10.068 per 100,000 population (EAPC = −3.503%). Japan also saw decreases in both case numbers and incidence rates. In Europe, Bulgaria experienced a decrease in both case numbers and incidence rates, while Finland's incidence rate remained relatively stable with little change. In the Americas, Brazil reported a significant decline in both case numbers and incidence rates, and the United States also showed a downward trend. In Africa, Egypt exhibited an increase in case numbers but a decrease in incidence rate, a pattern similarly observed in Nigeria. These regional differences emphasize the diverse epidemiological patterns of ICH among WCBA, as reflected in case numbers, incidence rates, and EAPC trends (Supplementary Table S1; Figure 2).

#### 3.3.2 Mortality

In 2021, China recorded the highest number of ICH-related deaths among WCBA globally, with an estimated 15,276.876 deaths (95% uncertainty interval [UI]: 11,349.704–19,877.692). Meanwhile, Nauru had the highest mortality rate, at 40.063 per 100,000 population (95% UI: 25.431–62.654; Supplementary Table S2; Supplementary Figures S2A, B). Between 1990 and 2021, Zimbabwe experienced the largest increase in mortality rate, with an estimated annual percentage change (EAPC) of 5.125% (95% CI: 3.590%−6.683%), whereas Slovenia saw the greatest decline, with an EAPC of −9.321% (95% CI: −9.788% to −8.851%; Supplementary Table S2; Supplementary Figure S2C). In 2021, the global mortality rate of ICH among WCBA was 4.628 per 100,000 population (95% UI: 4.059–5.237), which was higher than that of 115 countries but lower than 89 countries worldwide (Table 2; Supplementary Table S2).

Mortality trends varied across regions: In Asia, China's number of deaths decreased from 27,523.589 in 1990 to 15,276.876 in 2021, with the mortality rate dropping from 10.712 to 3.742 per 100,000 population (EAPC = −3.467%). Japan also experienced declines in both death count and mortality rate.

In Europe, Belgium showed a significant reduction in both mortality rate and number of deaths, whereas Finland saw minimal change in mortality. In the Americas, Brazil recorded a substantial decrease in both mortality rate and death count, while the United States also exhibited a declining trend. In Africa, Egypt reported a decrease in both mortality rate and death count (EAPC = −3.323%), while Nigeria initially saw a drop in deaths followed by an increase, though its mortality rate continued to decline (EAPC = −3.175%; Supplementary Table S2; Supplementary Figure S2).

#### 3.3.3 DALYs

In 2021, China reported the highest number of ICH-related DALYs among WCBA globally, with an estimated 826,125.230 DALYs (95% UI: 635,586.424–1,049,381.326). At the same time, Nauru recorded the highest DALY rate, at 2,102.100 per 100,000 population (95% UI: 1,351.553–3,263.003; Supplementary Table S3; Supplementary Figures S3A, B). Between 1990 and 2021, Zimbabwe experienced the largest increase in DALY rate, with an EAPC of 5.003% (95% CI: 3.524%−6.503%), while the Maldives saw the most significant decline, with an EAPC of −7.322% (95% CI: −7.659% to −6.984%; Supplementary Table S3; Supplementary Figure S3C). In 2021, the global DALY rate for ICH among WCBA was 249.334 per 100,000 population (95% UI: 219.195–279.770), making it higher than in 114 countries but lower than in 90 countries worldwide (Table 2; Supplementary Table S3).

Regional variations in DALY trends were notable. In Asia, both China and Japan experienced declines in DALY counts and rates. Specifically, in China, the DALY count dropped from 1,491,269.652 in 1990 to 826,125.230 in 2021, while the DALY rate decreased from 561.818 to 210.394 per 100,000 population (EAPC = −3.280%). In Europe, Belgium demonstrated a marked decline in both DALY count and rate, while Finland showed relatively minor changes. In the Americas, Brazil experienced a significant reduction in both DALY count and rate, while the United States also saw a decline, though to a lesser extent. In Africa, Egypt reported reductions in both DALY count and rate, while Nigeria initially experienced a decrease in DALY count followed by an increase, though its DALY rate continued to decline (Supplementary Table S3; Supplementary Figure S3).

### 3.4 GBD at the regional and country levels

From 1990 to 2021, the incidence rate, mortality rate, and disability-adjusted life years (DALYs) of ICH among WCBA showed a global decline across all regions, though with notable regional variations. Tropical Latin America (a middle SDI region) experienced the most significant reduction in incidence. Oceania, High-income North America, and the Caribbean saw slower declines, with these regions mostly concentrated in mid-to-high SDI categories.

Central Europe (a high-middle SDI region) had the largest decline in mortality, whereas Oceania, High-income Asia Pacific, and the Caribbean showed slower reductions. Central Europe also recorded the most significant decline in DALYs, while Oceania, Eastern Europe, High-income North America, and the Caribbean exhibited slower decreases, again mostly within mid-to-high SDI regions. Conversely, some low- and middle-income regions saw a rise in incidence rates, particularly in Oceania and Western Sub-Saharan Africa, both of which fall under low and middle SDI regions (Supplementary Figure S8).

### 3.5 Frontier analysis

**Figure 4:**
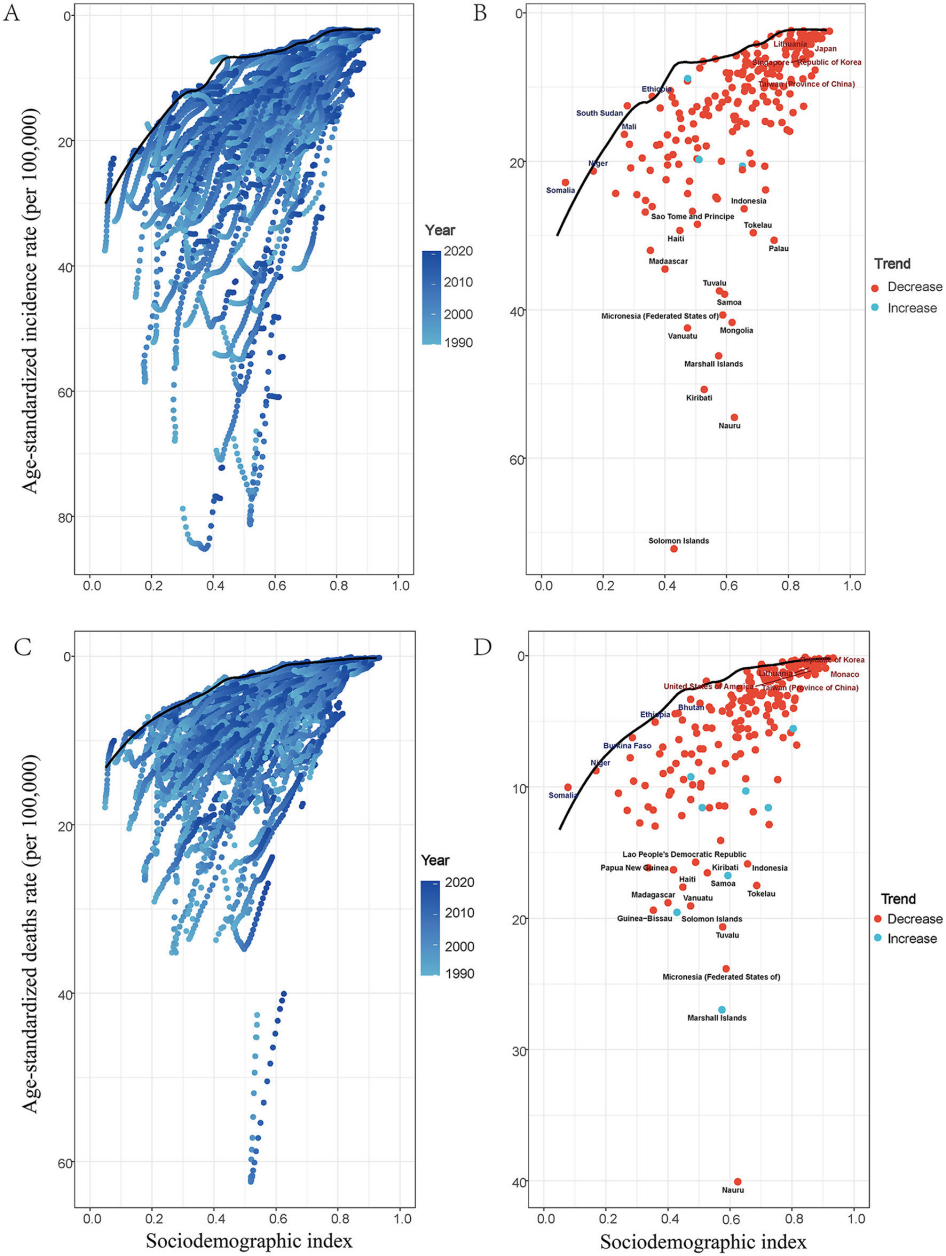
Frontier analysis exploring the relationships of SDI with ASDR and with ASIR for ICH among WCBA in 204 countries and territories. In figures **(A)**, **(C)**, the color change from light blue (1990) to dark blue (2021) represents the change in years. In figures **(B)**, **(D)**, each point represents specific country or territory in 2021, the frontier line is shown in black, and the top 15 countries and territories with the largest differences from the frontier are marked in black. Blue represents low-SDI with the smallest differences from the frontier, red represents high-SDI with the largest differences from the frontier. The direction of ASDR and ASIR change from 1990 to 2021 is indicated by the color of the dots, with orange dots representing decreases and green dots representing increases.

Using data from 1990 to 2021, a frontier analysis examined the relationship between Social Development Index (SDI), age-standardized mortality rate (ASDR), and age-standardized incidence rate (ASIR) of ICH among WCBA in 204 countries and regions (Figure 4). As is shown in Panels A and C, over time, low SDI regions had more dispersed data points, with some areas experiencing an increase in ASIR, while others showed minor changes or slight decreases, leading to an inconsistent and fluctuating trend. In middle SDI regions, data points were more concentrated, with most countries showing a slight upward trend in ASIR over time, though the magnitude varied. High SDI regions exhibited a general downward trend in later years, though some areas experienced increases.

Regarding ASDR, low-SDI regions exhibited a similar pattern, with scattered data points showing an increase in ASDR in many regions, while others experienced little change or a decline. Middle-SDI regions displayed a gradual increase in ASDR, though the magnitude varied, presenting a more consistent trend compared to low-SDI regions. In contrast, high-SDI regions generally showed a declining ASDR trend, although some countries experienced an increase.

Figure B and D highlight that by 2021, some high-SDI countries, such as South Korea and China, experienced a declining trend in ASDR, while certain low-SDI regions, including Mongolia and the Federated States of Micronesia, exhibited varied ASDR trends.

These findings underscore significant differences in the burden and trends of ICH among WCBA across countries and regions with different SDI levels. They also highlight the inconsistencies in ASIR and ASDR trends within each SDI category, suggesting that multiple factors influence ICH incidence and mortality, rather than SDI levels alone.

### 3.6 Inequality analysis

**Figure 5:**
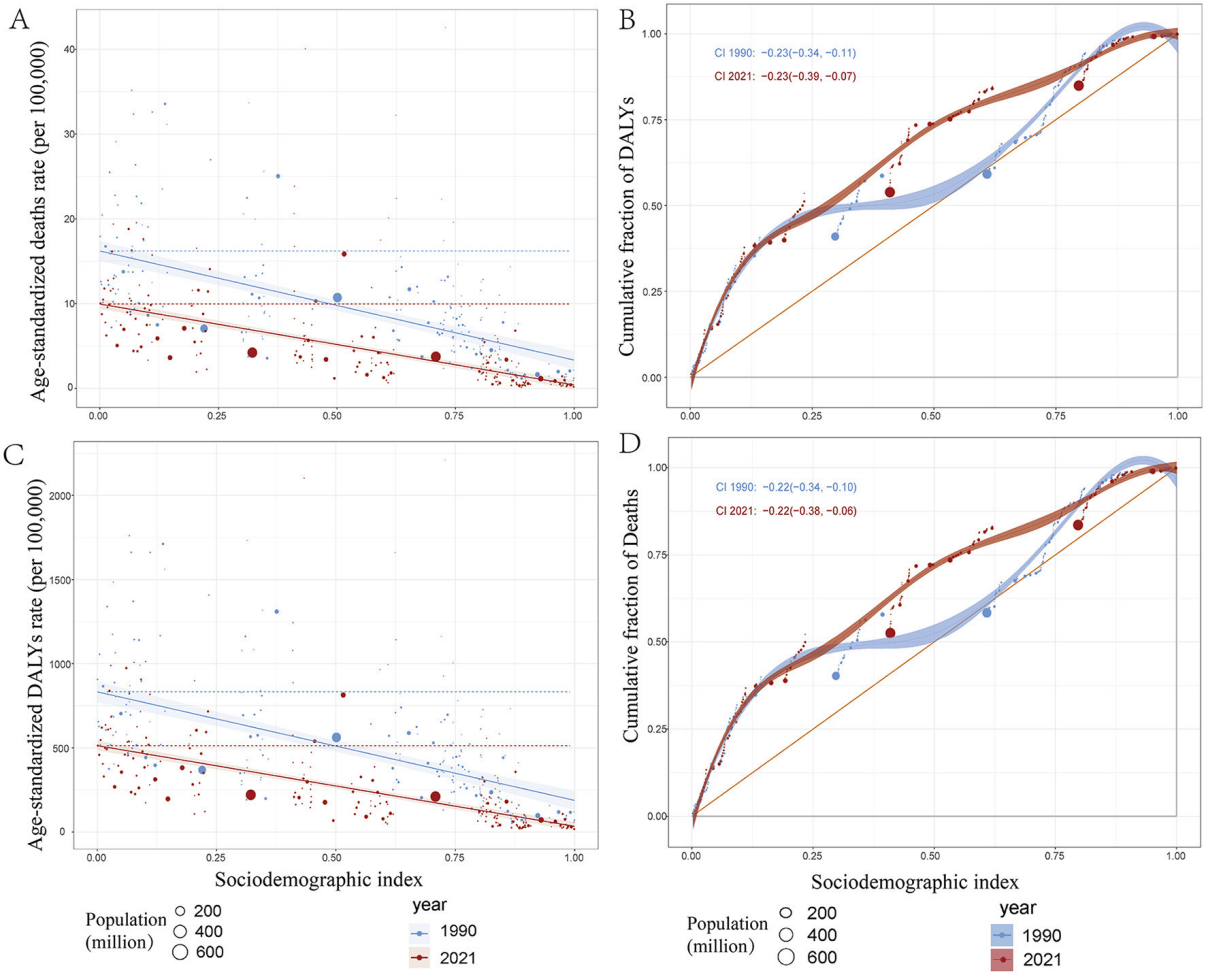
Health inequality regression curves and concentration curves for the deaths and disability—adjusted life—years (DALYs) of ICH among WCBA in 1990 and 2021. **(A, C)** illustrate the slope index of inequality, showing the relationship between the Socio—demographic Index (SDI) and age—standardized death rates, as well as age—standardized DALYs rates for each condition. The points represent individual countries and territories, and their sizes are scaled by population. **(B, D)** present the concentration index, which quantifies relative inequalities by integrating the area under the Lorenz curve, aligning the distribution of deaths and DALYs with the population distribution by SDI. Blue represents data from 1990, and red represents data from 2021. DALYs: disability—adjusted life—years.

Analysis of mortality and disability-adjusted life year (DALY) burdens associated with ICH among WCBA revealed significant absolute and relative inequalities linked to Social Development Index (SDI), showing a negative correlation. Countries with lower SDI levels generally experienced a higher burden of mortality and DALYs (Figures 5A, C). In 1990, low-SDI countries exhibited substantially higher mortality rates and DALY burdens, with regression curves indicating strong inequality. By 2021, this inequality had lessened, as evidenced by a reduction in burden among low-SDI countries (highlighted by red data points in the panel). The slope of the regression curve declined from 1990 to 2021, suggesting a narrowing of health disparities. In 1990, mortality and DALYs were highly concentrated in low-SDI regions. By 2021, the regression curve had shifted closer to the equality line (45-degree line), indicating a decline in relative inequality (Figures 5B, D). Although low-SDI regions still bear a significant burden, these findings suggest that global health disparities have somewhat narrowed between 1990 and 2021. However, despite improvements in high-SDI countries, low-SDI regions continue to face substantial health challenges.

### 3.7 Risk factors

The Global Burden of Disease (GBD) 2021 study employed a systematic comparative risk assessment framework to identify and quantify 26 risk factors contributing to ICH burden among WCBA. This framework, built upon systematic reviews and meta-analyses, followed established causal inference criteria including biological plausibility and evidence consistency. The GBD 2021 hierarchical classification organized risk factors into five categories: (1) metabolic risks (*n* = 8), including elevated blood pressure and fasting plasma glucose; (2) environmental and occupational risks (*n* = 7), such as ambient particulate matter and lead exposure; (3) behavioral risks (*n* = 6), including tobacco use and physical inactivity; (4) air pollution cluster (*n* = 3), encompassing ambient and occupational particulate exposures; and (5) other risks (*n* = 2), including intimate partner violence (14).

To address potential overlap among risk factors, the GBD framework utilized mediation analysis and the joint population attributable fraction (PAF) formula—Joint PAF = 1 – ∏(1 – PAF_*i*_)—to prevent double counting and prioritize proximal risk factors (25, 26). High systolic blood pressure emerged as the most significant contributor, accounting for 3.03% of ICH-related deaths and 153.35% of attributable DALYs in WCBA. This was followed by metabolic risks (2.87% deaths, 146.48% DALYs), air pollution (2.14% deaths, 110.43% DALYs), environmental/occupational risks (1.74% deaths, 89.45% DALYs), and behavioral risks (1.3% deaths, 67.32% DALYs). Between 1990 and 2020, most risk factors showed declining trends, with substantial reductions in air pollution, high systolic blood pressure, and household air pollution from solid fuels. However, ambient particulate matter pollution and high body mass index exhibited increasing trends (Supplementary Figure S10).”

### 3.8 BAPC model projections of ICH among WCBA

Using a Bayesian Age-Period-Cohort (BAPC) model, projections were made for the age-standardized incidence rate (ASIR) and age-standardized death rate (ASDR) of ICH among WCBA from 2022 to 2035. The model predicts a continued decline in both ASIR and ASDR (Supplementary Figure S11). ASIR is expected to decrease from 5.69 per 100,000 population in 2021 (95% UI: 5.66–5.70) to 4.77 per 100,000 in 2035 (95% UI: 4.52–5.02), representing a 16.2% reduction; ASDR is projected to decline from 2.29 per 100,000 in 2021 (95% UI: 2.28–2.29) to 1.77 per 100,000 in 2035 (95% UI: 1.71–1.83), reflecting a 22.8% reduction (Supplementary Tables S4, S5). These projected declines are likely driven by advancements in healthcare and improved risk factor management. However, the widening uncertainty intervals in later years underscore the need to monitor demographic shifts and track emerging lifestyle-related risk factors. These factors could influence the future burden of ICH among WCBA, highlighting the importance of sustained public health interventions.

## 4 Discussion

Intracerebral Hemorrhage (ICH), a sudden-onset cerebrovascular event characterized by high rates of disability and mortality, represents a critical global health concern for women of childbearing age (WCBA). This condition significantly compromises women's quality of life, undermines family wellbeing, and imposes substantial burdens on healthcare systems and society (3, 14). This study represents the first comprehensive analysis of the global burden of ICH in WCBA, utilizing data from the Global Burden of Disease Study 2021 (GBD 2021) spanning 1990 to 2021.

Our findings reveal a global decline in ICH burden among WCBA over the past three decades, evidenced by reductions in age-standardized incidence, mortality, and disability-adjusted life years (DALYs). However, these improvements have been unevenly distributed across regions, with high-Socio-demographic Index (SDI) regions experiencing the most pronounced declines, while low-SDI regions—particularly Sub-Saharan Africa and South Asia—continue to bear disproportionately high burdens. Among risk factors, elevated systolic blood pressure emerged as the predominant contributor to ICH-related mortality and DALYs, while environmental factors such as air pollution and metabolic risks including diabetes exhibited complex temporal trends (27).

These findings align with prior regional studies reporting declining global stroke mortality, although most have focused on general stroke populations rather than WCBA specifically (28, 29). The identification of hypertension as the leading modifiable risk factor corroborates evidence from clinical cohort studies in WCBA (30). The magnitude of global ICH burden decline reported here exceeds that of previous single-country analyses, possibly reflecting enhanced global surveillance and reporting mechanisms. The persistence of regional disparities corresponds with World Health Organization reports on health equity, though few large-scale studies have quantified ICH burden variation between high- and low-SDI regions (31). Discrepancies between our global findings and some localized studies reporting stable or increasing ICH incidence may reflect methodological differences in age standardization and case ascertainment between global epidemiological models and regional clinical studies (32).

The pronounced geographic disparities underscore the urgent need for targeted public health interventions in resource-limited settings. Given hypertension's role as the leading modifiable risk factor, scalable and sustainable blood pressure screening and management programs could significantly reduce ICH burden among WCBA. The observed decrease in traditional risk factors such as household air pollution from solid fuels, alongside rising threats including ambient particulate matter and obesity, necessitates adaptive public health strategies. The notably higher burden in WCBA aged 45–49 years highlights the importance of integrating ICH risk assessment into family planning and preconception care, as cerebrovascular risks may influence fertility decisions and contraceptive choices.

Several limitations warrant acknowledgment. First, GBD estimates lack clinical validation specific to pregnancy-associated ICH, which may involve distinct pathophysiological mechanisms and outcomes due to unique vascular changes during pregnancy and the postpartum period. Second, underreporting in low-income countries with limited healthcare infrastructure may underestimate true ICH burden, potentially obscuring deeper global health inequities. Third, the standardized GBD methodology cannot capture population-specific risk factors such as pregnancy complications, peripartum cardiomyopathy, or genetic susceptibility. Fourth, the inherent time lag of GBD data (typically 1–2 years) limits timely evaluation of intervention effects and emerging epidemiological patterns.

Future research should prioritize clinical validation of GBD estimates among WCBA, particularly in under-resourced regions. Prospective, multicenter cohort studies incorporating pregnancy-specific risk factors, genetic biomarkers, and detailed clinical phenotypes are essential for improved risk stratification. Implementation studies evaluating the effectiveness of scalable interventions—such as enhanced hypertension control and reduced environmental exposures—in low-SDI regions are crucial for informing evidence-based policymaking.

## 5 Conclusion

National health policies should integrate ICH prevention into maternal health frameworks, focusing on healthcare infrastructure development in under-resourced areas and advancing population-level cardiovascular risk reduction strategies. International collaboration is essential for addressing global health inequities and achieving Sustainable Development Goals related to non-communicable diseases. These findings provide critical evidence to inform targeted, evidence-based strategies for policymakers, clinicians, and public health practitioners aiming to reduce ICH burden and improve health outcomes among WCBA globally.

## Data Availability

The datasets presented in this study can be found in online repositories. The names of the repository/repositories and accession number(s) can be found in the article/Supplementary material.

## Supplementary material

Supplementary Table S1 Incidence of Intracerebral Hemorrhage among Women of Childbearing age, by Countries and Territories, 1990-2021.

Supplementary Table S2 Mortality of Intracerebral Hemorrhage among Women of Childbearing age, by Countries and Territories, 1990-2021.

Supplementary Table S3 Disability-adjusted life years (DALYs) of Intracerebral Hemorrhage among Women of Childbearing age, by Countries and Territories, 1990-2021.

Supplementary Table S4 Forecast of the Incidence Rate of Intracerebral Hemorrhage among Women of Childbearing Age from 1990 to 2035.

Supplementary Table S5 Forecast of the Mortality Rate of Intracerebral Hemorrhage among Women of Childbearing Age from 1990 to 2035.

Supplementary Figure S1 Visualization of GBD 2021 Workflow.

Supplementary Figure S2 Mortality of ICH among WCBA across 204 countries and territories.A, Number of deaths cases. B, Deaths rate. C, Estimated annual percentage change (EAPC) in Mortality.

Supplementary Figure S3 Disability-adjusted life years (DALYs) of ICH among WCBA across 204 countries and territories. A, Number of DALYs cases. B, DALYs rate. C, Estimated annual percentage change (EAPC) in DALYs.

Supplementary Figure S4 Age-specific percentages of ICH among WCBA incidence, mortality, and disability-adjusted life years (DALYs) in 2021. A, Incidence. B. Deaths. C, DALYs.

Supplementary Figure S5 This figure illustrates the trends in age - standardized incidence (A), mortality (B), and disability—adjusted life—years (DALYs) (C) rates of ICH among WCBA across 204 countries and territories, categorized by the Socio - Demographic Index (SDI) from 1990 to 2021. For each individual country and territory, the points plotted from left to right represent the estimates for each year within the 1990–2021 time frame. The blue line indicates the average expected correlation between the SDI and the burden - related rates of ICH among WCBA. This correlation is calculated based on the data from each geographical region throughout the 1990–2021 estimation period. In this context, DALYs denote disability - adjustted life - years, and SDI represents the Socio - Demographic Index.

Supplementary Figure S6 This figure illustrates the trends in age - standardized incidence (A), mortality (B), and disability - adjusted life - years (DALYs) (C) rates of ICH among WCBA across 204 countries and territories, categorized by the Socio - Demographic Index (SDI) from 1990 to 2021. For each individual country and territory, the points plotted from left to right represent the estimates for each year within the 1990 - 2021 time fram. The blue line indicates the average expected correlation between the SDI and the burden - related rates of ICH among WCBA. This correlation is calculated based on the data from each geographical region throughout the 1990 - 2021 estimation period. In this context, DALYs denote disability - adjusted life - years, and SDI represents the Socio - Demographic Index.

Supplementary Figure S7 Trends in incidence, mortality, and disability - adjusted life years (DALYs) of ICH among WCBA by Regional Level, 1990–2021. A: Incidence rate. B: Mortality rate. C: DALYs rate.

Supplementary Figure S8 Temporal trend of ICH among WCBA incidence, mortality, and disability-adjusted life years (DALYs) from 1990 to 2021. A, Incidence. B. Deaths. C, DALYs.

Supplementary Figure S9 Risk Factors Contributing to the Global Burden of ICH among WCBA. A. Mortality Rates; B. DALY Rates.

Supplementary Figure S10 Trends in Global Burden and Risk Factors of ICH among WCBA (1990–2020): A. Mortality Rates; B. DALY Rates.

Supplementary Figure S11 Incidence Rate per 100,000 population uture Forecasts of tthe Global Burden of ICH among WCBA. A: Age - Standardized Incidence Rate (ASIR), B: Age - Standairdized Death Rate (ASDR).
