# Supplementary figures and images for: Global, regional, and national epidemiology of Intracerebral Hemorrhage among women of childbearing age (1990–2021): incidence, mortality, DALYs, and risk factor analysis
Source: Front Neurol. 2025 Aug 5;16:1602507. doi: 10.3389/fneur.2025.1602507 (PMC12361251; doi:10.3389/fneur.2025.1602507)

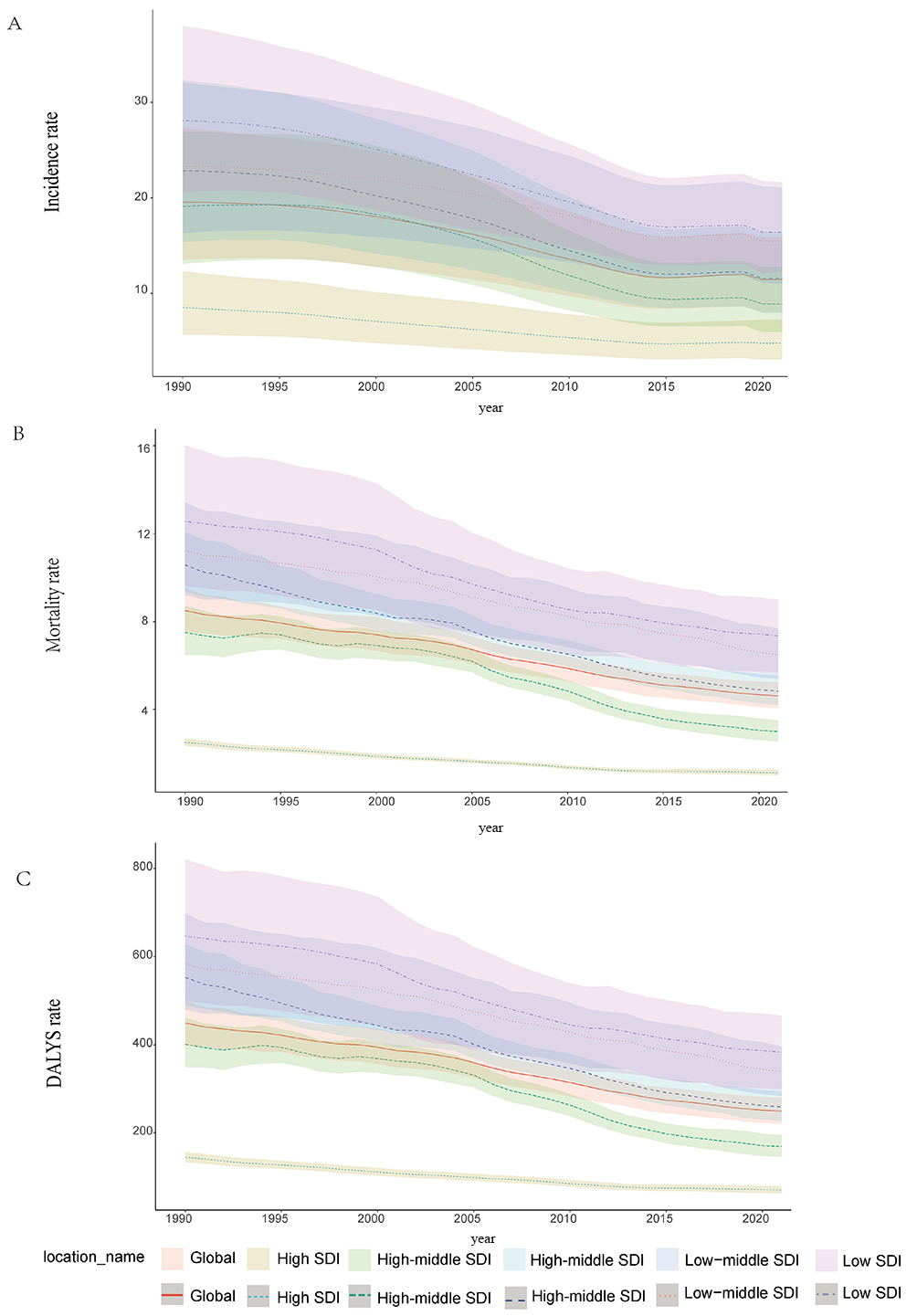

Supplement: Supplementary Figure S1 — Visualization of GBD 2021 Workflow. [file Data_Sheet_1.zip › Figure S7.tif]

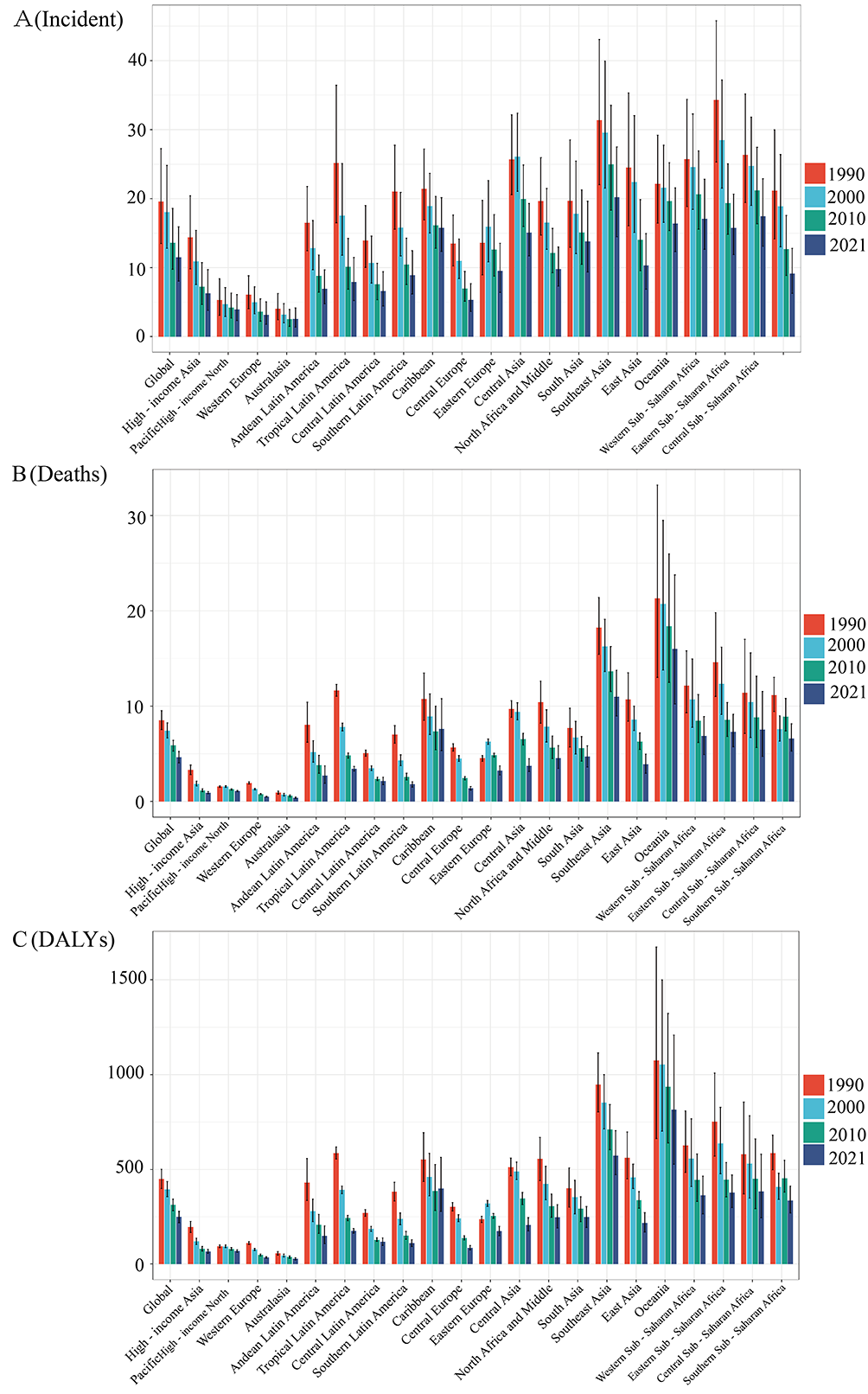

Supplement: Supplementary Figure S1 — Visualization of GBD 2021 Workflow. [file Data_Sheet_1.zip › Figure S8.tif]

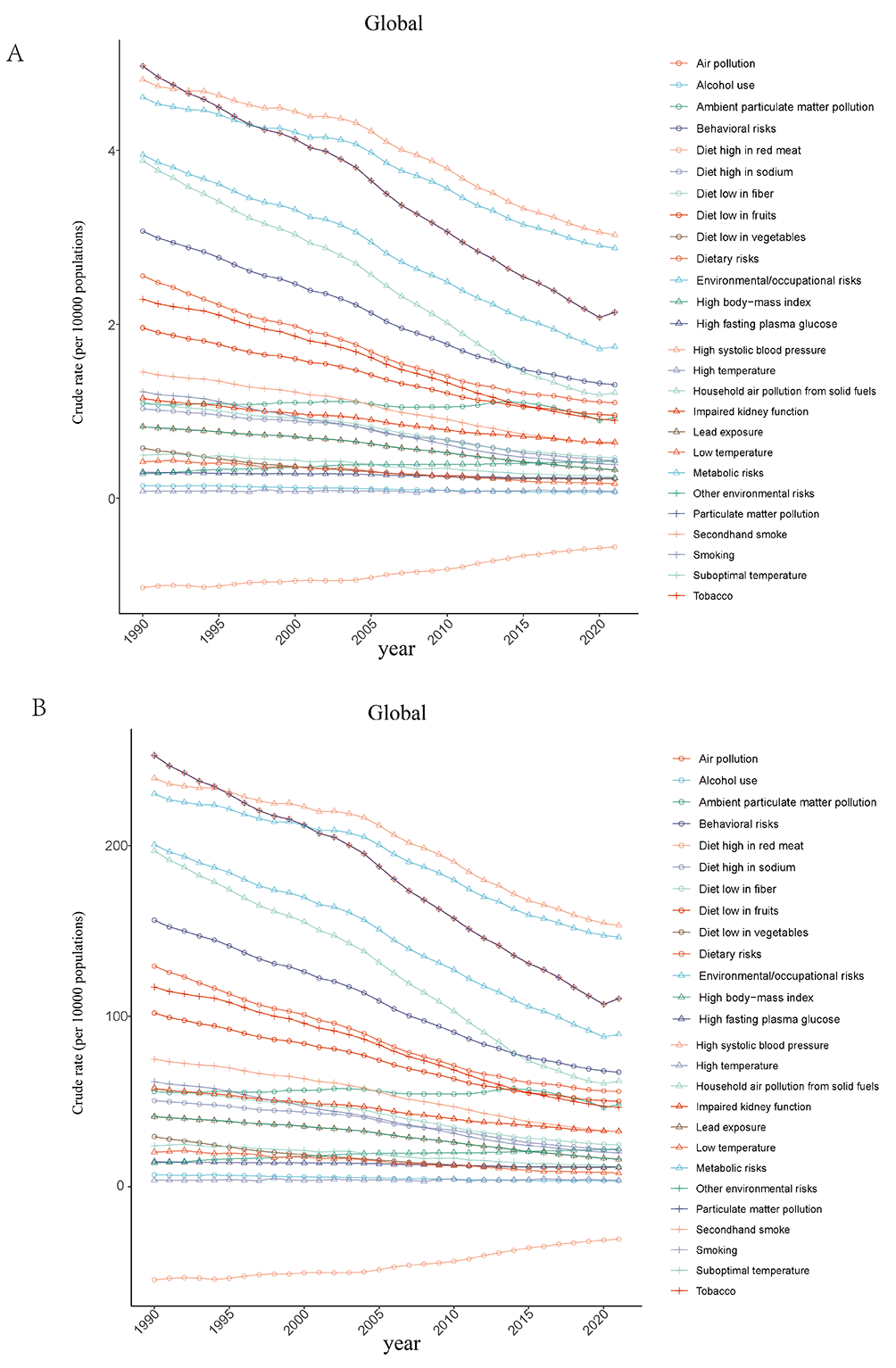

Supplement: Supplementary Figure S1 — Visualization of GBD 2021 Workflow. [file Data_Sheet_1.zip › Figure S9.tif]

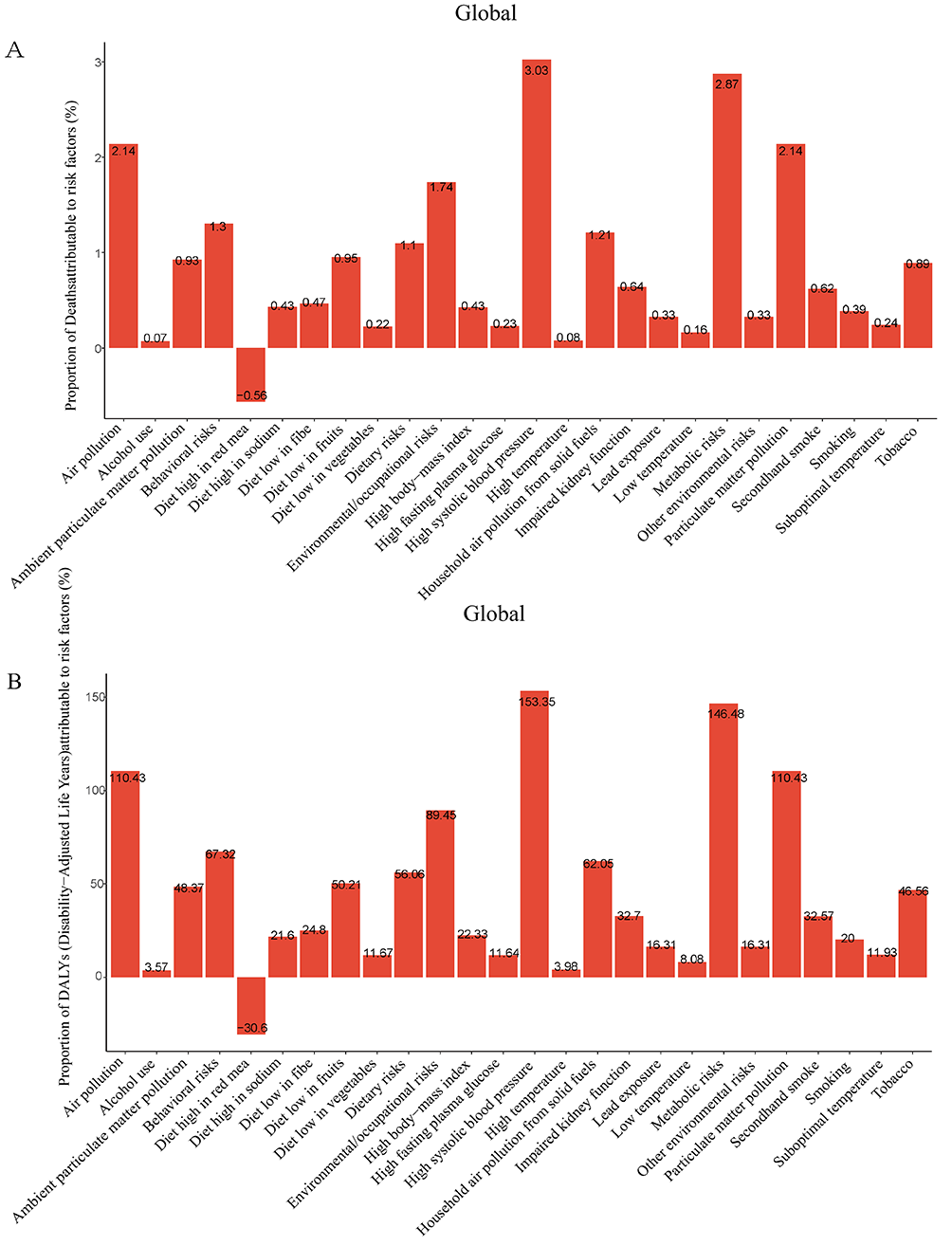

Supplement: Supplementary Figure S1 — Visualization of GBD 2021 Workflow. [file Data_Sheet_1.zip › Figure S10.tif]

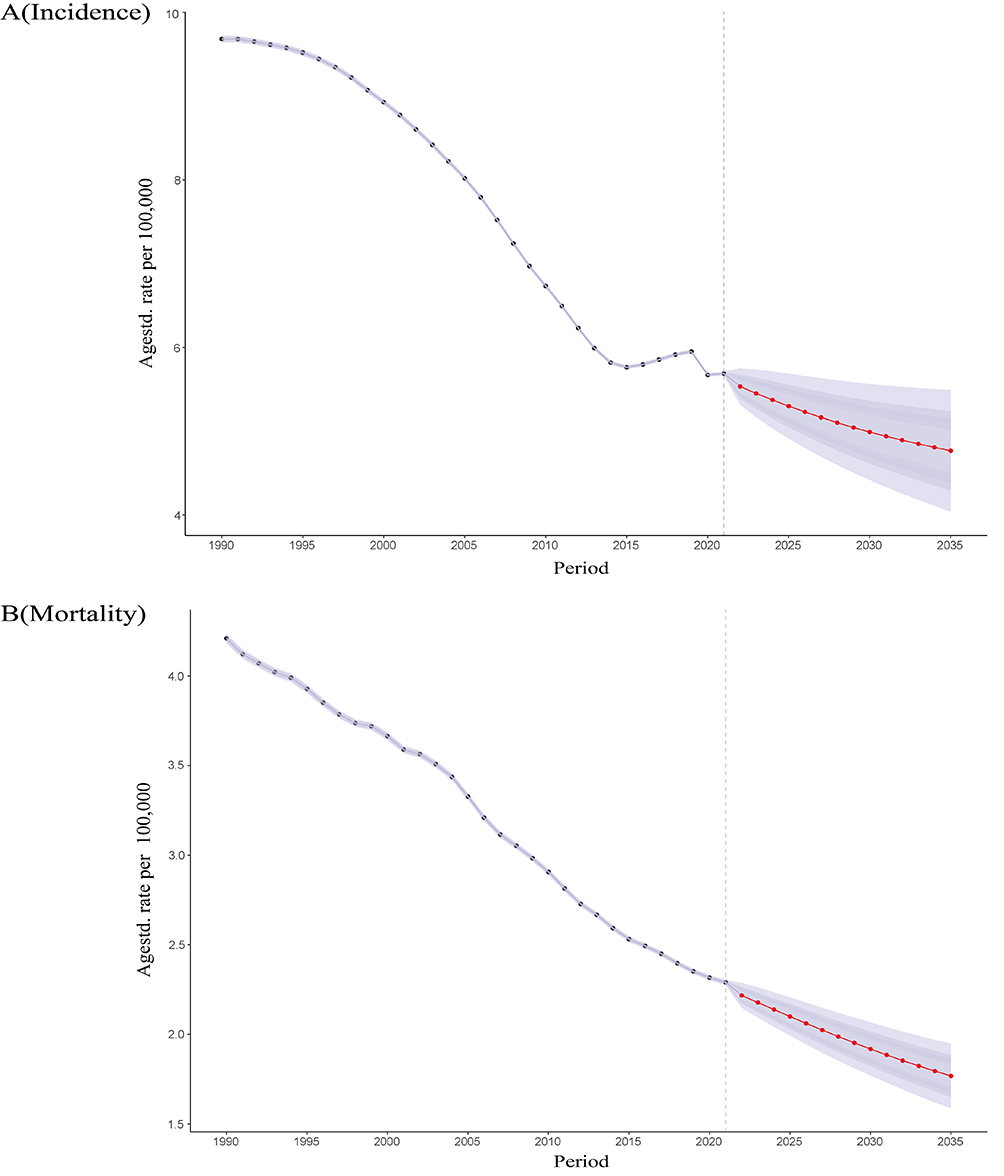

Supplement: Supplementary Figure S1 — Visualization of GBD 2021 Workflow. [file Data_Sheet_1.zip › Figure S11.tif]

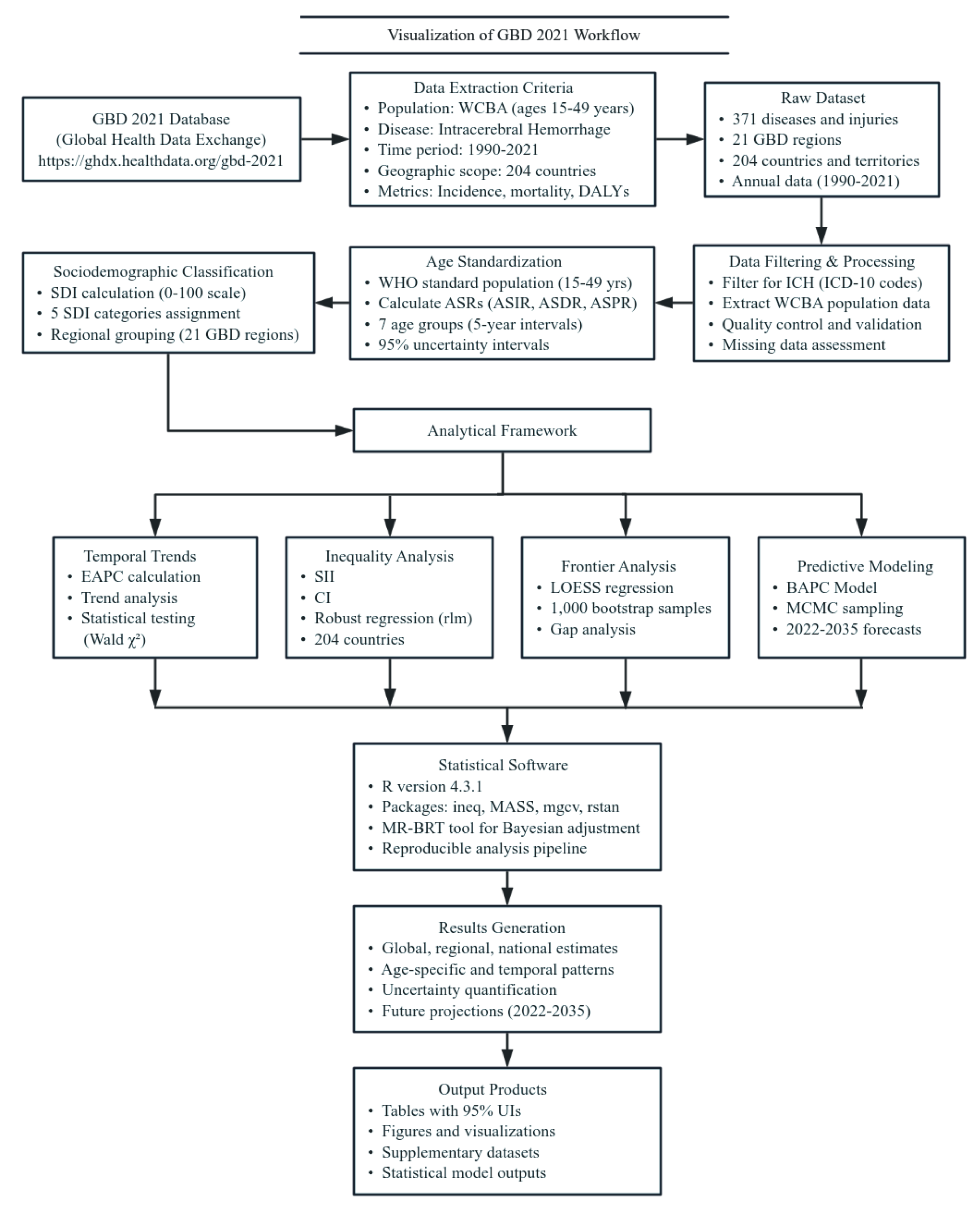

Supplement: Supplementary Figure S1 — Visualization of GBD 2021 Workflow. [file Data_Sheet_1.zip › Figure S1.tif]

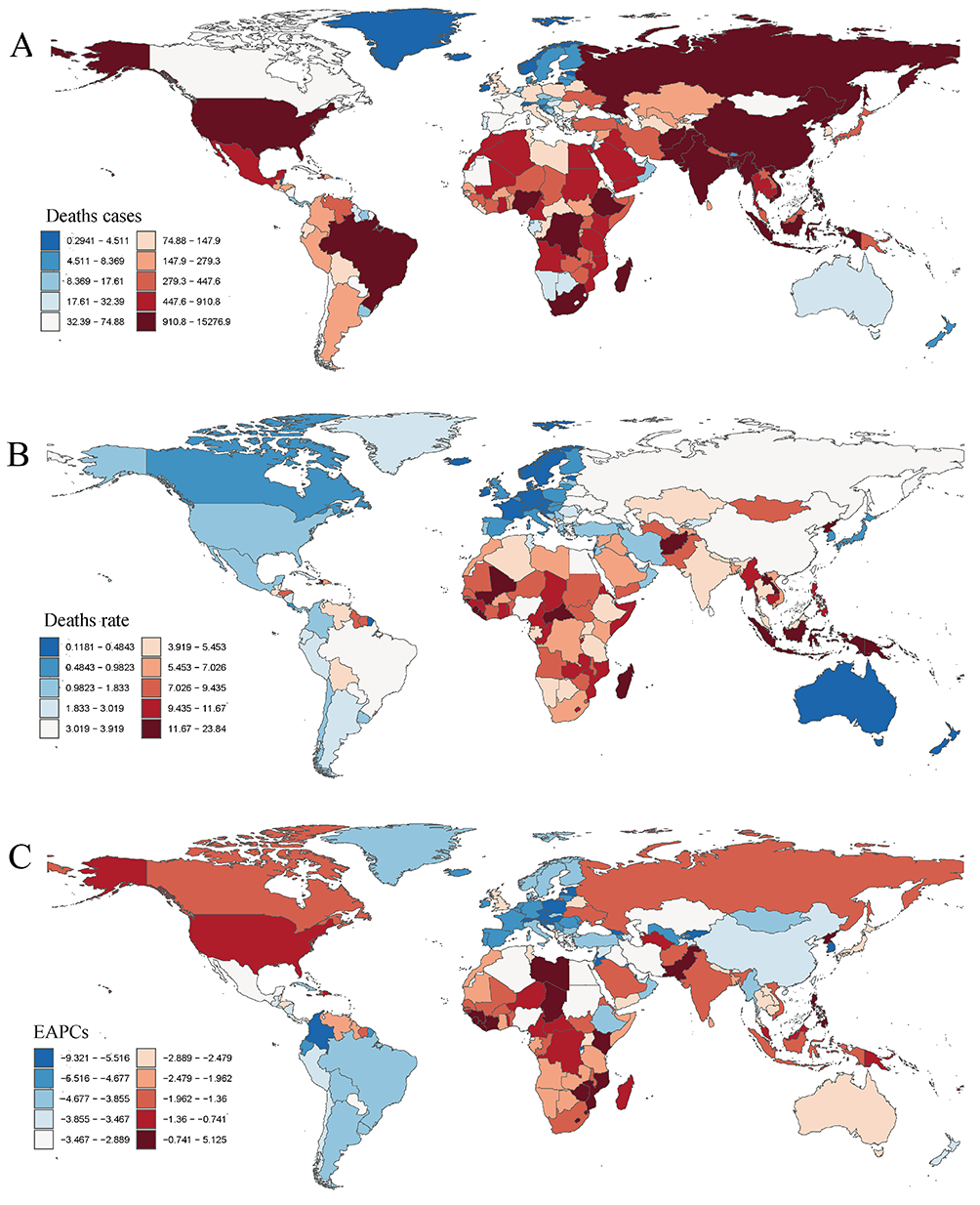

Supplement: Supplementary Figure S1 — Visualization of GBD 2021 Workflow. [file Data_Sheet_1.zip › Figure S2.tif]

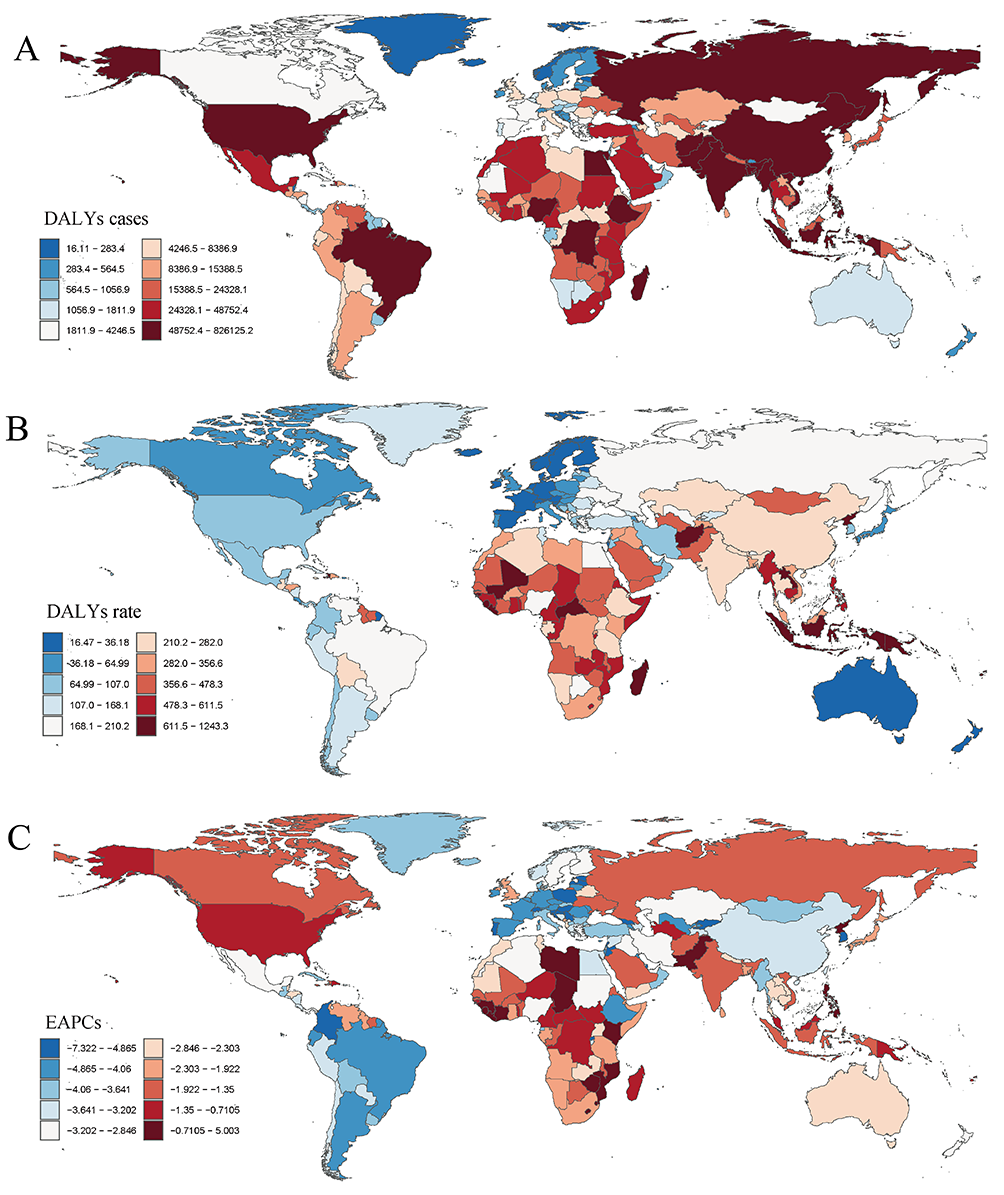

Supplement: Supplementary Figure S1 — Visualization of GBD 2021 Workflow. [file Data_Sheet_1.zip › Figure S3.tif]

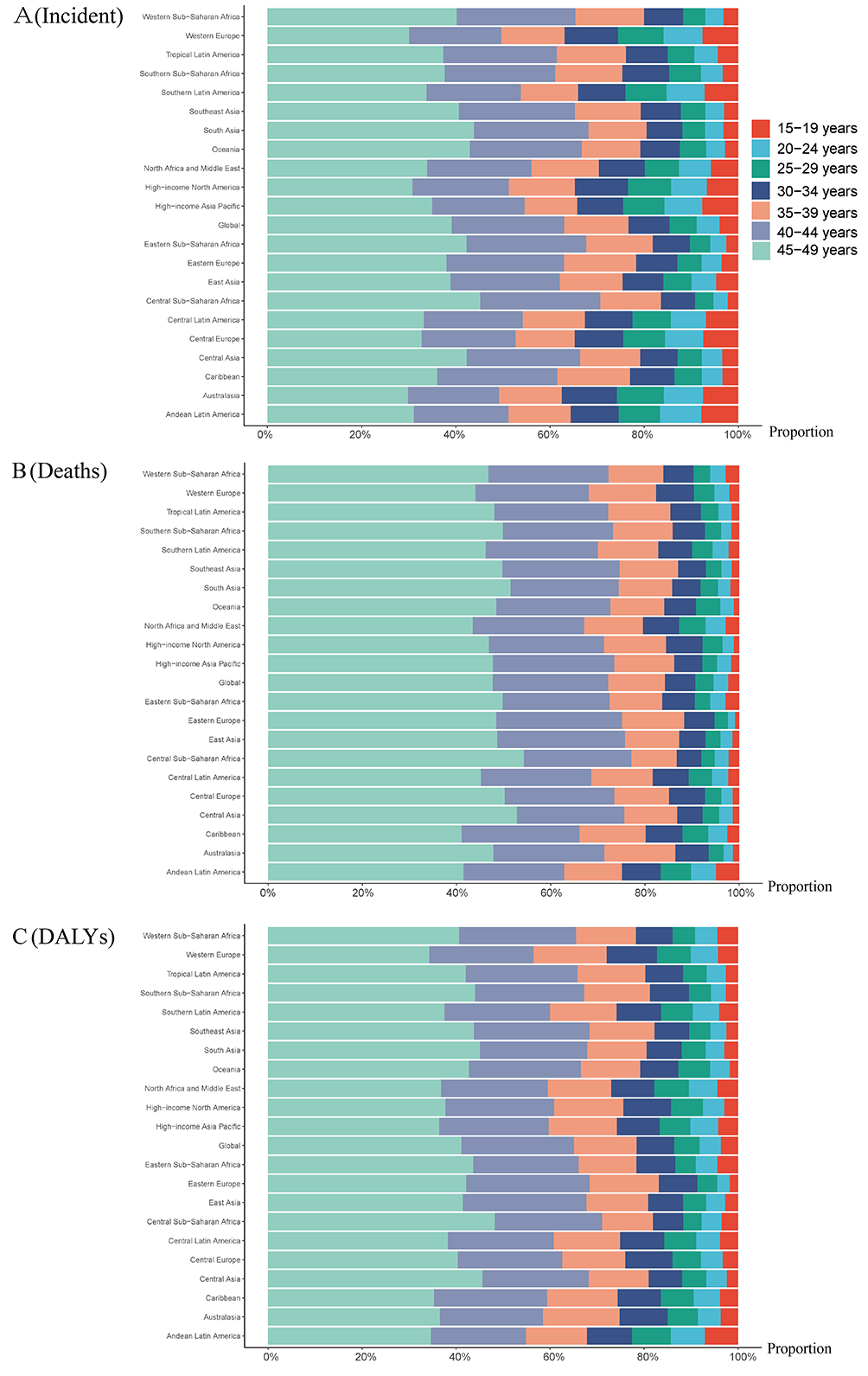

Supplement: Supplementary Figure S1 — Visualization of GBD 2021 Workflow. [file Data_Sheet_1.zip › Figure S4.tif]

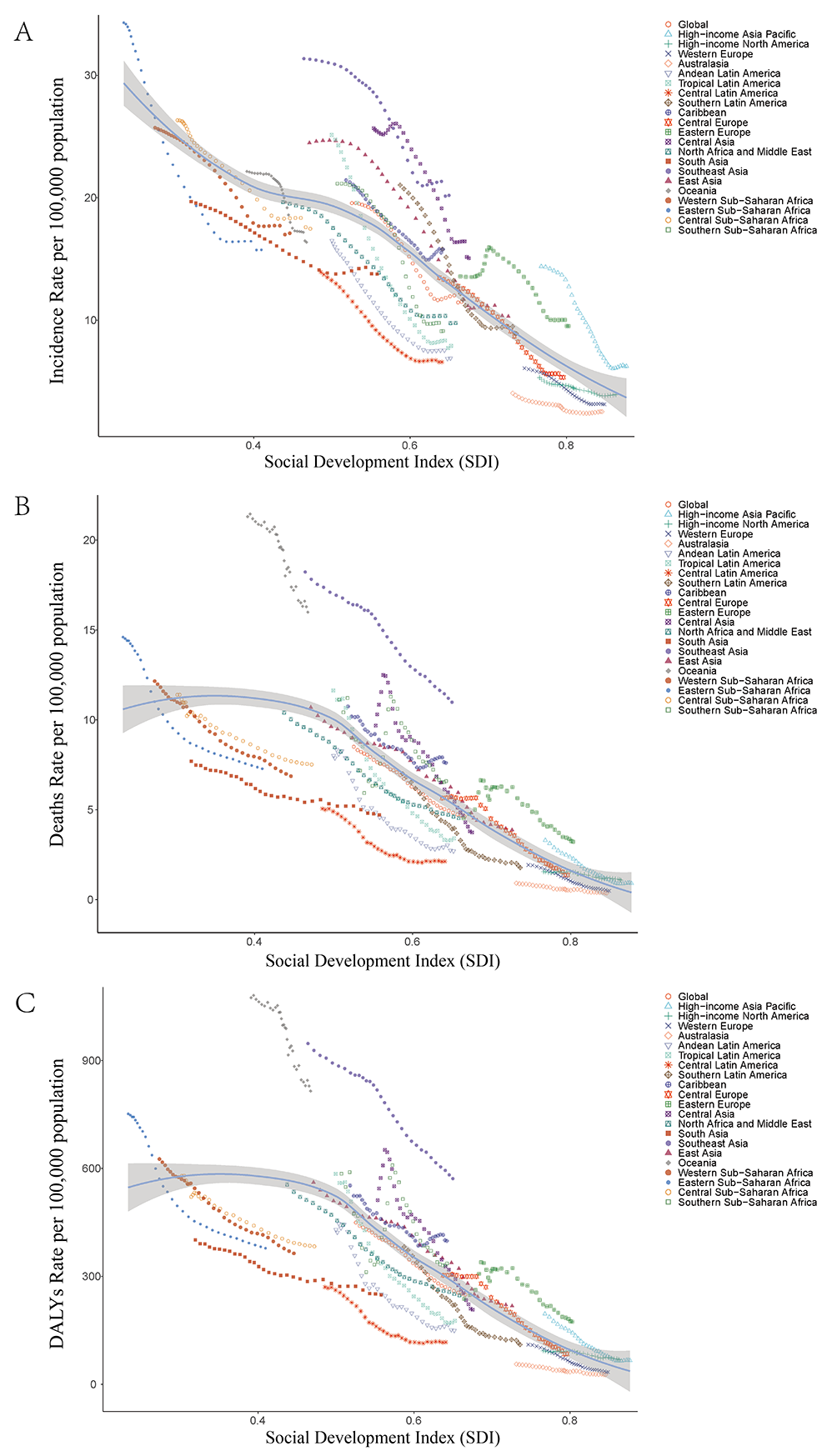

Supplement: Supplementary Figure S1 — Visualization of GBD 2021 Workflow. [file Data_Sheet_1.zip › Figure S5.tif]

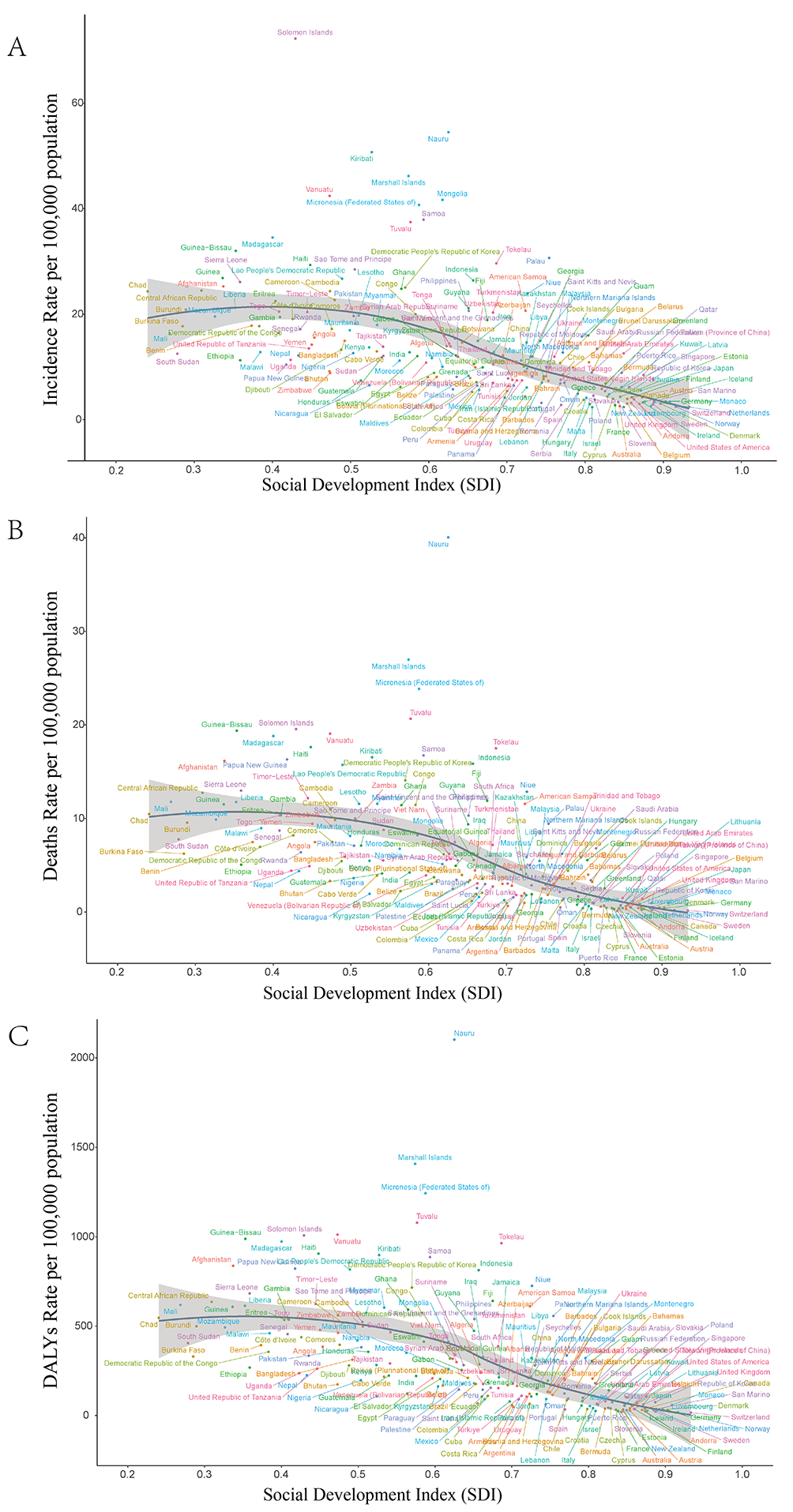

Supplement: Supplementary Figure S1 — Visualization of GBD 2021 Workflow. [file Data_Sheet_1.zip › Figure S6.tif]
